# Provenance-and machine learning-based recommendation of parameter values in scientific workflows

**DOI:** 10.7717/peerj-cs.606

**Published:** 2021-07-05

**Authors:** Daniel Silva Junior, Esther Pacitti, Aline Paes, Daniel de Oliveira

**Affiliations:** 1 Institute of Computing, Universidade Federal Fluminense, Niteroi, RJ, Brazil; 2 Inria, CNRS, LIRMM, University of Montpellier, Montpellier, France

**Keywords:** Scientific workflows, Recommender systems, Machine Learning, Preference Learning

## Abstract

Scientific Workflows (SWfs) have revolutionized how scientists in various domains of science conduct their experiments. The management of SWfs is performed by complex tools that provide support for workflow composition, monitoring, execution, capturing, and storage of the data generated during execution. In some cases, they also provide components to ease the visualization and analysis of the generated data. During the workflow’s composition phase, programs must be selected to perform the activities defined in the workflow specification. These programs often require additional parameters that serve to adjust the program’s behavior according to the experiment’s goals. Consequently, workflows commonly have many parameters to be manually configured, encompassing even more than one hundred in many cases. Wrongly parameters’ values choosing can lead to crash workflows executions or provide undesired results. As the execution of data- and compute-intensive workflows is commonly performed in a high-performance computing environment e.g., (a cluster, a supercomputer, or a public cloud), an unsuccessful execution configures a waste of time and resources. In this article, we present *FReeP—Feature Recommender from Preferences*, a parameter value recommendation method that is designed to suggest values for workflow parameters, taking into account past user preferences. *FReeP* is based on Machine Learning techniques, particularly in Preference Learning. *FReeP* is composed of three algorithms, where two of them aim at recommending the value for one parameter at a time, and the third makes recommendations for n parameters at once. The experimental results obtained with provenance data from two broadly used workflows showed *FReeP* usefulness in the recommendation of values for one parameter. Furthermore, the results indicate the potential of *FReeP* to recommend values for n parameters in scientific workflows.

## Introduction

Scientific experiments are the basis for evolution in several areas of human knowledge (De Oliveira, Liu & Pacitti, 2019; Mattoso et al., 2010; Hey & Trefethen, 2020; Hey, Gannon & Pinkelman, 2012). Based on observations of open problems in their research areas, scientists formulate hypotheses to explain and solve those problems (Gonçalves & Porto, 2015). Such hypothesis may be confirmed or refuted, and also can lead to new hypotheses. For a long time, scientific experiments were manually conducted by scientists, including instrumentation, configuration and management of the environment, annotation and analysis of results. Despite the advances obtained with this approach, time and resources were wasted since a small misconfiguration of the parameters of the experiment could compromise the whole experiment. The analysis of errors in the results was also far from trivial (De Oliveira, Liu & Pacitti, 2019).

The evolution in computer science field allowed for the development of technologies that provided useful support for scientists in their experiments. One of these technologies is *Scientific Workflows* (De Oliveira, Liu & Pacitti, 2019; Deelman et al., 2005). A scientific workflow (named *workflow* henceforth) is an abstraction that represents each step of the experiment expressed as an *activity*, which has input data and relationships (i.e., data dependencies) with other activities, according to the stages of the experiment (Zhao & Ioan Raicu, 2008).

Several workflows commonly require the execution of multiple data-intensive operations as loading, transformation, and aggregation (Mattoso et al., 2010). Multiple computational paradigms can be used for the design and execution of workflows, e.g., shell and Python scripts (Marozzo, Talia & Trunfio, 2013), Big Data frameworks (e.g., Hadoop and Spark) (Guedes et al., 2020b), but they are usually managed by complex engines named Workflow Management Systems (WfMS). A key feature that a WfMS must address is the efficient and automatic management of parallel processing activities in High Performance Computing (HPC) environments (Ogasawara et al., 2011). Besides managing the execution of the workflow in HPC environments, WfMSs are also responsible for capturing, structuring and recording metadata associated to all the data generated during the execution: input data, intermediate data, and the final results. These metadata is well-known as *provenance* (Freire et al., 2008). Based on provenance data, it is possible to analyze the results obtained and to foster the reproducibility of the experiment, which is essential to prove the veracity of a produced result.

In this article, the concept of an experiment is seen as encompassing the concept of a workflow, and not as a synonym. A workflow may be seen as a controlled action of the experiment. Hence, the workflow is defined as one of the *trials* conducted in the context of an experiment. In each trial, the scientist needs to define the parameter values for each activity of the workflow. It is not unusual that a simple workflow has more than 100 parameters to set. Setting up these parameters may be simple for an expert, but not so simple for non-expert users. Although WfMSs represent a step forward by providing the necessary infrastructure to manage workflow executions, they provide a little help (or even no help at all) on defining parameter values for a specific workflow execution. A good parameters values tune in a workflow execution is crucial not only for the quality of the results but also influences if a workflow will execute or not (avoiding unnecessary execution crashes). A poor choice of parameters values can cause failures, which leads to a waste of execution time. Failures caused by poor choices of parameter values are even more severe when workflows are executing in HPC environments that follow a pay-as-you-go model, e.g., clouds, since they can increase the overall financial cost.

This way, if the WfMS could “learn” from previous successfully executions of the workflow and recommend parameter values for scientists, some failures could be avoided. This recommendation is especially useful for non-expert users. Let us take as an example a scenario where an expert user has modeled a workflow and executed several trials of the same workflow varying the parameter values. If a non-expert scientist wants to execute the same workflow with a new set of parameter values and input data, but does not know how to set the values of some of the parameters, one can benefit from parameter values used on previous executions of the same (or similar) workflow. The advantage of the WfMS is provenance data already contains the parameter values used on previous (successful) executions and can be a rich resource to be used for recommendation. Thus, this article hypothesis is that *by adopting an approach to recommend the parameters values of workflows in a WfMS, we can increase the probability that the execution of workflow will be completed*. As a consequence, the financial cost associated with execution failures is reduced.

In this article, we propose a method named *FReeP—Feature Recommender From Preferences*, which aims at recommending values for parameters of workflow activities. The proposed approach is able to recommend parameter values in two ways: (i) a single parameter value at a time, and (ii) multiple parameter values at once. The proposed approach relies on user preferences, defined for a subset of workflow parameters, together with the provenance of the workflow. It is essential to highlight that user preferences are fundamental to explore experiment variations in a scientific scenario. Furthermore, for our approach, user preferences help prune search space and consider user restrictions, making personalized recommendations. The idea of combining user preferences and provenance is novel and allows for producing a personalized recommendation for scientists. FReeP is based on Machine Learning algorithms (Mitchell, 2015), particularly, Preference Learning (Fürnkranz & Hüllermeier, 2011), and Recommender Systems (Ricci, Rokach & Shapira, 2011). We evaluated *FReeP* using real workflow traces (considered as benchmarks): Montage (Hoffa et al., 2008) from astronomy domain and SciPhy (Ocaña et al., 2011) from bioinformatics domain. Results indicate the potential of the proposed approach. This article is an extension of the conference paper “FReeP: towards parameter recommendation in scientific workflows using preference learning” (Silva Junior et al., 2018) published in the Proceedings of the 2018 Brazilian Symposium on Databases (SBBD). This extended version provides new empirical shreds of evidence regarding several workflow case studies as well as a broader discussion on related work and experiments.

This article is organized in five sections besides this introduction. “Background” section details the theoretical concepts used in the proposal development. “FReeP—Feature Recommender from Preferences” section presents the algorithm developed for the problem of parameters value recommendation using user preferences. “Experimental Evaluation” section shows the results of the experimental evaluation of the approach in three different scenarios. Then, “Related Work” section presents a literature review with papers that have addressed solutions to problems related to the recommendation applied to workflows and the Machine Learning model hyperparameter recommendation. Lastly, “Conclusion” section brings conclusions about this article and points out future work.

## Background

This section presents key concepts for understanding the approach presented in this article to recommend values for parameters in workflows based on users’ preferences and previous executions. Initially, it is explained about scientific experiments. Following, the concepts related to Recommender Systems are presented. Next, the concept of *Preference Learning* is presented. This section also brings a *Borda Count* overview, a non-common voting schema that is used to decide which values to suggest to the user.

### Scientific experiment

A scientific experiment arises from the observation of some phenomena and questions raised from the observation. The next step is the hypotheses formulation aiming at developing possible answers to those questions. Then, it is necessary to test the hypothesis to verify if an output produced is a possible solution. The whole process includes many iterations of refinement, consisting, for example, of testing the hypothesis under distinct conditions, until it is possible to have enough elements to support it.

The scientific experiment life-cycle proposed by Mattoso et al. (2010) is divided into three major phases: *composition*, *execution* and *analysis*. The *composition* phase is where the experiment is designed and structured. *Execution* is the phase where all the necessary instrumentation for the accomplishment of the experiment must be finished. Instrumentation means the definition of input data, parameters to be used at each stage of the experiment, and monitoring mechanisms. Finally, the *analysis* phase is when the data generated by the composition and execution phases are studied to understand the obtained results. The approach presented in this article focus on the *Execution* phase.

### Scientific workflows

*Scientific workflows* have become a de facto standard for modeling in silico experiments (Zhou et al., 2018). A *Workflow* is an abstraction that represents the steps of an experiment and the dataflow through each of these steps. A workflow is formally defined as a directed acyclic graph *W*(*A*,*Dep*). The nodes }{}A = \{ {a_1},{a_2}, \ldots ,{a_n}\} are the activities and the edges *Dep* represent the data dependencies among activities in *A*. Thus, given *a*_*i*_ :amp:mid; (1 ≤ *i* ≤ *n*), the set *P* = {*p*_1_, *p*_2_, …, *p*_*m*_} represents the possible input parameters for activity *a*_*i*_ that defines the behavior of *a*_*i*_. Therefore, a *workflow* can be represented as a graph where the vertices act as experiment steps and the edges are the relations, or the dataflow between the steps.

A workflow can also be categorized according to the level of abstraction into *conceptual* or *concrete*. A conceptual workflow represents the highest level of abstraction, where the experiment is defined in terms of steps and dataflow between them. This definition does not explain how each step of the experiment will execute. The concrete *workflow* is an abstraction where the activities are represented by the computer programs that will execute them. The execution of an activity of the workflow is called an *activation* (De Oliveira et al., 2010b), and each activation invokes a program that has its parameters defined. However, managing this execution, which involves setting the correct parameter values for each program, capturing the intermediate data and execution results, becomes a challenge. It was with this in mind, and with the help of the composition of the experiment in the workflow format, that *Workflow Management Systems (WfMS)*, such as Kepler (Altintas et al., 2006), Pegasus (Deelman et al., 2005) and SciCumulus (De Oliveira et al., 2010b) emerged.

In special, SciCumulus is a key component of the proposed approach since it provides a framework for parallel workflows to benefit from FReeP. Also, data used in the experiments presented in this article are retrieved from previous executions of several workflows in SciCumulus. It is worth noticing that other WfMSs such as Pegasus and Kepler could also benefit from FReeP as long as they provide necessary provenance data for recommendation. SciCumulus architecture is modularized to foster maintainability and ease the development of new features. SciCumulus is open-source and can be obtained at https://github.com/UFFeScience/SciCumulus/. The system is developed using MPI library (a de facto standard library specification for message-passing), so SciCumulus is a distributed application, i.e., each SciCumulus module has multiple instances created on the machines of the distributed environment (which are different processes and each process has multiple threads) that communicate, triggering functions for sending and receiving messages between these processes. According to Guerine et al. (2019), SciCumulus has four main modules: (i) *SCSetup*, (ii) *SCStarter*, (iii) *SCCore*, and (iv) *SCQP* (SciCumulus Query Processor). The first step towards executing a workflow in SciCumulus is to define the workflow specification and the parameters values to be consumed. This is performed using the SCSetup module. The user has to inform the structure of the workflow, which programs are associated to which activities, *etc*. When the metadata related to the experiment is loaded into the SciCumulus database, the user can start executing the workflow. Since SciCumulus was developed focusing on supporting the execution of workflows in clouds, instantiating the environment was a top priority. The SCSetup module queries the provenance database to retrieve prospective provenance and creates the virtual machines (in the cloud) or reserve machines (in a cluster). The SCStarter copies and invokes an instance of SCCore in each machine of the environment, and since SCCore is a MPI-based application it runs in all machines simultaneously and follows a Master/Worker architecture (similar to Hadoop and Spark). The SCCore-Master (SCCore_0_) schedules the activations for several workers and each worker has a specific ID (SCCore_1_, SCCore_2_, *etc*.). When a worker is idle, it sends a message for the SCCore_0_ (Master) and request more activations to execute. The SCCore_0_ defines at runtime the best activation to send following a specific cost model. The SCQP component allows for users to submit queries to the provenance database for runtime or *post-mortem* analysis. For more information about SciCumulus please refer to (De Oliveira et al., 2012; De Oliveira et al., 2010a; Guerine et al., 2019; Silva et al., 2020; Guedes et al., 2020a; De Oliveira et al., 2013).

### Provenance

An workflow activation has input data, and generates intermediate and output data. WfMS has to collect all metadata associated to the execution in order to foster reproducibility. This metadata is called *provenance* (Freire et al., 2008). According to Goble (2002), the provenance must verify data quality, path audit, assignment verification, and information querying. Data quality check is also related to verifying the reliability of workflow generated data. Path audit is the ability to follow the steps taken at each stage of the experiment that generated a given result. The assignment verification is linked to the ability to know who is responsible for the data generated. Lastly, an information query is essential to analyze the data generated by the experiment’s execution. Especially for workflows, provenance can be classified as prospective (p-prov) and retrospective (r-prov) (Freire et al., 2008). *p-prov* represents the specification of the workflow that will be executed. It corresponds to the steps to be followed to achieve a result. *r-prov* is given by executed activities and information about the environment used to produce a data product, consisting of a structured and detailed history of the execution of the workflow.

*Provenance* is fundamental for the scientific experiment analysis phase. It allows for verifying what caused an activation to fail or generated an unexpected result, or in the case of success, what were the steps and parameters used until the result. Another advantage of provenance is the reproducibility of an experiment, which is essential for the validation of the results obtained by third parties. Considering the provenance benefits in scientific experiments, it was necessary to define a model of representation of provenance (Bose, Foster & Moreau, 2006). The standard W3C model is *PROV* (Gil et al., 2013). *PROV* is a generic data model and is based on three basic components and their links, being the components: *Entity*, *Agent* and *Activity*. The provenance and provenance data model are essential concepts because *FReeP* operation relies on provenance to recommend parameter values. Also, to extract provenance data to use in *FReeP* it is necessary to understand the provenance data model used.

### Recommender systems

*FReeP* is a personalized *Recommender system (RS)* (Resnick & Varian, 1997) aiming at suggesting the most relevant parameters to the user to perform a task, based on their preferences.There are three essential elements for the development of a recommender system: *Users*, *Items*, and *Transactions*. The *Users* are the target audience of the recommender system with their characteristics and goals. *Items* are the recommendation objects and *Transactions* are records that hold a tuple *(user, interaction)*, where the interaction encompasses the actions that the user performed when using the recommender system. These interactions are generally user feedbacks, which may be interpreted as their preferences.

A recommender task can be defined as: given the elements *Items*, *Users* and *Transactions*, find the most useful items for each user. According to Adomavicius & Tuzhilin (2005), a recommender system must satisfy the equation \forall u \in U,{{i}^{\prime}_u} = arg{\kern 1pt} ma{x_{i \in I}}F(u,i), where *U* represents the users, *I* represents the items and *F* is a utility function that calculates the utility of an item *i in I* for a *u in U* user. In case the tuple (*u*, *i*) is not defined in the entire search space, the recommender system can extrapolate the *F* function.

**Figure 1:**
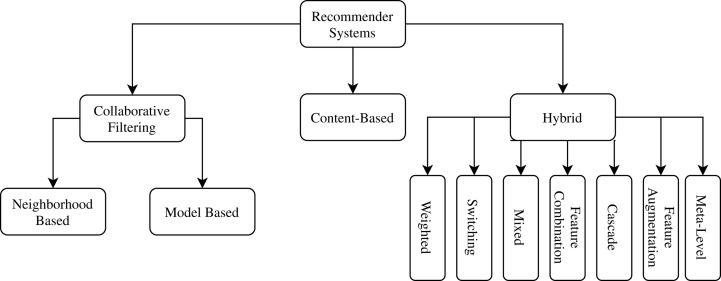
Related types of recommender systems taxonomy.

The utility function varies according to the approach followed by the recommender system. Thus, recommender systems are categorized according to the different strategies used to define the utility function. The most common approaches to recommender systems are: *Content Based, Collaborative Filtering* and *Hybrids*. Figure 1 provides a taxonomy related types of recommender systems for this work.

In *Collaborative Filtering Recommender Systems*, a recommendation is based on other users’ experience with items in the system domain. The idea is related to the human behavior of, at times, giving credit to another person’s opinion about what should be done in a given situation. The *Neighborhood Based* subtype strictly follows the principle that users with similar profiles have similar preferences. The *Model-Based* subtype generates a hypothesis from the data and use it to make recommendations instantly. Although widely adopted, Collaborative Filtering only uses collective information, limiting novel discoveries in scientific experiment procedures.

*Content-based Recommender Systems* make recommendations similar to items that the user has already expressed a positive rating in the past. To determine the similarity degree between items, this approach is highly dependent on extracting their characteristics. However, each scenario needs the right item representation to give satisfactory results. In scientific experiments, it can be challenging to find an optimal item representation.

Finally, *Hybrid Recommender Systems* arise out of an attempt to minimize the weaknesses that traditional recommendation techniques have when used individually. Also, it is expected that a hybrid strategy can aggregate the strengths of the techniques used together. There are several methods of combining recommendation techniques in creating a hybrid recommender system, including: *Weighting* approaches that provides a score for each recommendation item, *Switching*, which allows for selecting different types of recommending strategies, *Mixing*, to make more than one recommentation at a time, *Feature Combination*, to put together both Content-Based and Collaborative Filtering strategies, *Cascade*, that first filters the candidate items for the recommendation, followed by refining these candidates, looking for the best alternatives, *Feature Augmentation*and *Meta-Level*, which chain a series of recommendations one after another (Burke, 2002).

*FReeP* is as a *Cascade Hybrid Recommender System* because the content of user preferences is used to prune the search space followed by a collaborative strategy to give the final recommendations.

### Preference learning

User preferences play a crucial role in recommender systems (Viappiani & Boutilier, 2009). From an Artificial Intelligence perspective, a *preference* is a problem restriction that allows for some degree of relaxation. Fürnkranz & Hüllermeier (2011) refers to Learning Preferences as “inducing preference models from empirical data”. In several scenarios, the empirical data is implicitly defined, for example, when the user’s preference is expressed by clicking on the most interesting products, instead of effectively buying one of them or stating that one is preferable over another.

A Preference Learning task consists of learning a predictive function that, given a set of items where preferences are already known, predicts preferences for a new set of items. The most common way of representing preferences is through binary relationships. For example, a tuple (*x*_*i*_ > *x*_*j*_) > would mean a preference for the value *i* over *j* for the attribute *x*.

The main task within Preference Learning area is *Learning to Rank* as commonly it is necessary to have an ordering of the preferences. The task is divided into three categories: *Label Ranking* (Vembu & Gärtner, 2011), *Instance Ranking* (Bergeron et al., 2008) and *Object Ranking* (Nie et al., 2005). In *Label ranking* a *ranker* makes an ordering of the set of classes of a problem for each instance of the problem. In cases where the classes of a problem are naturally ordered, the *instance ranking* task is more suitable, as it orders the instances of a problem according to their classes. The instances belonging to the “highest” classes precede the instances that belong to the “lower” classes. In *object ranking* an instance is not related to a class. This task’s objective is, given a subset of items referring to the total set of items, to produce a ranking of the objects in that subset—for example, the ranking of web pages by a search engine.

*Pairwise Label Ranking* (Fürnkranz & Hüllermeier, 2003; Hüllermeier et al., 2008) (PLR) relates each instance with a preference type *a* > *b*, representing that *a* is preferable to *b*. Then, a binary classification task is assembled where each example *a*, *b* is annotated with *a* is *a* is preferable over *b* and 0, otherwise. Then, a classifier *M a*, *b* is trained over such dataset to learn how to make the preference predictions which returns 1 as a prediction that *a* is preferable to *b* and 0 otherwise. Instead of using a single classifier that makes predictions between *m* classes, given a set *L* of *m* classes, there will be *m*(*m* − 1)/2 binary classifiers, where a classifier *M i*, *j* only predicts between classes *i*, *j in L*. Then, the strategy defined by PLR uses the prediction of each classifier as a vote and uses a voting system that defines an ordered list of preferences. Next, we give more details about how *FReeP* tackles the voting problem.

### *Borda* count

*Voting Theory* (Taylor & Pacelli, 2008) is an area of Mathematics aimed at the study of voting systems. In an election between two elements, it is fair to follow the majority criterion, that is, the winning candidate is the one that has obtained more than half of the votes. However, elections involving more than two candidates require a more robust system. *Preferential Voting* (Karvonen, 2004) and *Borda Count* (Emerson, 2013) are two voting schemas concerning the scenarios where there are more than two candidates. In *Preferential Voting*, voters elicit a list of the most preferred to the least preferred candidate. The elected candidate is the one most often chosen as the most preferred by voters.

*Borda Count* is a voting system in which voters draw up a list of candidates arranged according to their preference. Then, each position in the user’s preference list gets a score. In a list of *n* candidates, the candidate in the *i-th* position on the list receives the score *n* − *i*. To determine the winner, the final score is the sum of each candidate’s scores for each voter, and the candidate with the highest score is the elected one.

**Figure 2:**
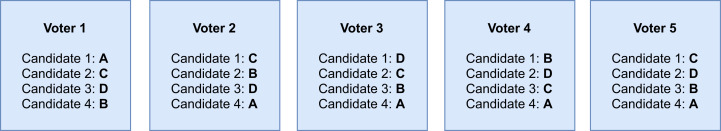
Votes example that each candidate received in voters preference order.

Figure 2 depicts an example of *Borda Count*. There are four candidates: *A*, *B*, *C* and *D*, and five vote ballots. The lines in each ballot represent the preference positions occupied by each candidate. As there are four candidates, the candidate preferred by a voter receives three points. The score for the candidate *D* is computed as follows: 1 voter elected the candidate *D* as the preferred candidate, then 1 * 3 = 3 points; 2 voters elected the candidate *D* as the second most preferred candidate, then 2 * 2 = 4 points; 2 voters elected the candidate *D* as the third most preferred candidate, then 2 * 1 = 2 points; 0 voters elected the candidate *D* as the least preferred, then 0 * 0 = 0 points. Finally, **candidate *D* total score** = 3 + 4 + 2 + 0 = 9.

Voting algorithms are used together with recommender systems to choose which items the users have liked best to make a good recommendation. Rani, Shokeen & Mullick (2017) proposed a recommendation algorithm based on clustering and a voting schema that after clustering and selecting the target user’s cluster, uses the *Borda Count* to select the most popular items in the cluster to be recommended. Similarly, Lestari, Adji & Permanasari (2018) compares *Borda Count* and the *Copeland Score* Al-Sharrah (2010) in a recommendation system based on Collaborative Filtering. Still using the *Borda Count*, Tang & Tong (2016) proposes the *BordaRank*. The method consists of using the *Borda Count* method directly in the sparse matrix of evaluations, without predictions, to make a recommendation.

## *FReeP—*Feature Recommender from Preferences

**Figure 3:**
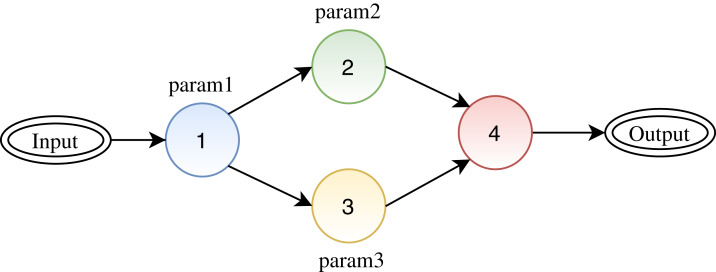
A synthetic workflow: circles represent activities, arrows between the circles represent the link between activities (data dependencies), and the labels for each circle represent the configuration parameters for each activity.

Figure 3 depicts a synthetic workflow, where one can see four activities represented by colored circles where activities 1, 2, and 3 have one parameter each. To execute the workflow, it is required to define values for parameters 1, 2, and 3. Given a scenario where a user has not defined values for all parameters, *FReeP* targets at helping the user to define values for the missing parameters. For this, *FReeP* divides the problem into two sub-tasks: (1) recommendation for only one parameter at a time; (2) recommendation for *n* parameters at once. The second task is more challenging than the first as parameters of different activities may present some data dependencies.

Taking into account user preferences, *FReeP* suggests parameter values that maximize a probability to make the workflow execute flawlessly until its end. *FReeP* receives a user preferences set to yield the personalized recommendations. The recommendations are the output of a model induced by a Machine Learning technique. *FReeP* is a hybrid recommendation technique as it incorporates aspects of both Collaborative Filtering and Content-Based concepts.

The way *FReeP* tackles the recommendation task is presented in three versions. In the first two versions, the algorithm aims at recommending a value for only one parameter at a time. While the naive version assumes that all parameters have a discrete domain, the enhanced second version is an extension of the first one that is able to deal with cases where a parameter has a continuous domain. The third version targets at recommending values for *n* > 1 parameters at a time.

Next, we start by presenting the naive version of the method that makes the recommendation for a single parameter at a time. Then, we follow to the improved version with enhancements that improve the performance and allows for working with parameters in the continuous domain. Finally, a generic version of the algorithm is presented, aiming at making the recommendation of values for multiple parameters at a time.

### Discrete domain parameter value recommendation

Given a provenance database *D*, a parameter *y* ∈ *Y*, where *Y* is the workflow parameters set, and a preferences or restrictions set *P* defined by the user, where *p*_*i*_ ∈ *P* (*y*_*i*_, *val*_*k*_), *FReeP* one parameter approach aims at solving the problem of recommending a *r* value for *y*, so that the *P* preferences together with the *r* recommendation to *y* maximize the chances of the workflow activation to run to the end.

**Figure 4:**
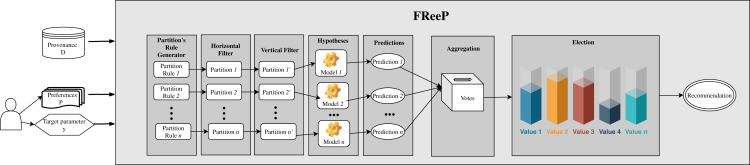
*FReeP* architecture overview.

Figure 4 presents an architecture overview of *FReeP*’s naive version. The algorithm receives as input the provenance database, a target workflow and user preferences. User preferences are also input as this article assumes that the user already has a subset of parameters for which has already defined values to use. In this naive version, the user preferences are only allowed in the form *a* = *b*, where *a* is a parameter, and *b* is a desired value to *a*.

Based on the user’s preferences, it would be possible to query the provenance database from which the experiment came from to retrieve records that could assist in the search for other parameters values that had no preferences defined. However, *FReeP* is based on a model generation that generalizes the provenance database, removing the user’s need to perform this query yet providing results that the query would not be able to return.

To obtain a recommendation from *FReeP*’s naive version, seven steps are required: **partitions generation**, **horizontal filter**, **vertical filter**, **hypothesis generation**, **predictions**, **aggregation**, and, finally **election-based recommendation**. Algorithm 1 shows the proposed algorithm to perform the parameter recommendation, considering the preferences for a subset or all other workflow execution parameters.

The algorithm input data are: target parameter for which the algorithm should make the recommendation, *y*; user preferences set, such as a list of key-values, where the key is a workflow parameter and value is the user’s preference for that parameter, *P*; provenance database, *D*.

The storage of provenance data for an experiment may vary from one WfMS to another. For example, SciCumulus, which uses a provenance representation derived from *PROV*, stores provenance in a relational database. Using SciCumulus example, it is trivial for the user responsible for the experiment to elaborate a SQL query that returns the provenance data related to the parameters used in each activity in a key-value representation. The key-value representation can be easily stored in a *csv* format file, which is the required format expected as provenance dataset in *FReeP* implementation. Thus, converting provenance data to the *csv* format is up to the user. Still, regarding the provenance data, the records present in the algorithm input data containing information about the parameters must be related only to executions that were successfully concluded, that is, there was no failure that resulted in the execution abortion. The inclusion of components to query and transform provenance data and force successful executions parameters selection would require implementations for each type of WfMS, which is out of the scope of this article.

**Figure 5:**
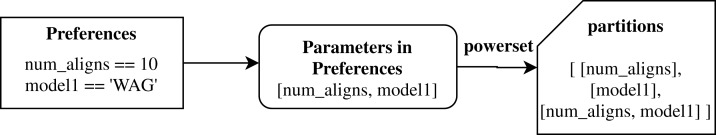
Example of *FReeP*’s partitioning rules generation for *Sciphy* provenance dataset using user’s preferences.

The initial step, **partitions_generation**, builds partitioning rules set based on the user’s preferences. Initially, the preference set parameters *P* are used to generate a *powerset*. This first step returns all generated *powerset* as a *partitions* ruleset. Figure 5 shows an example of how this first step works, with some parameters from *SciPhy* workflow.

**Figure 6:**
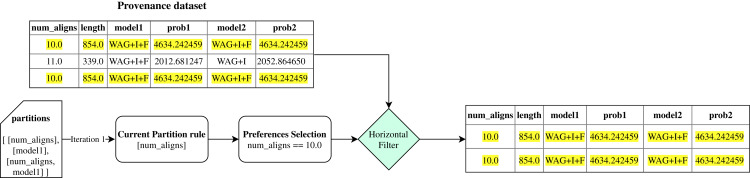
Example of *FReeP*’s horizontal filter using one partitioning rule for the *Sciphy* provenance dataset.

Then, FReeP initializes an iteration over the partitioning rules generated by the previous step. Iteration begins selecting only the records that follow the user’s preferences contained in the current ruleset, named in the algorithm as **horizontal_filter**. Figure 6 uses the *partitions* presented in Fig. 5 to show how the **horizontal_filter** step works.

**Figure 7:**
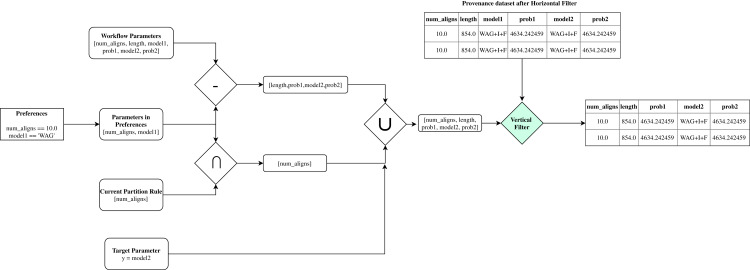
*FReeP*’s vertical filter step.

Subsequently, in the **vertical_filter** step, there is a parameter removal that aims at keeping only the recommendation target parameter, the parameters present in the current set of partitioning rules, and those that are neither the recommendation target parameter nor are present in any of the original user preferences. The last parameters mentioned remain because, in a next step, they can help to build a more consistent model. Thus, let *PW* be all workflow parameters set; *PP* the workflow parameters for which preference values have been defined; *PA* the parameters present in the partitioning rules of an iteration over the partitioning rules and *PV* = (*PW* − *PP*) ∪(*PP* ∩ *PA*) ∪ {*y*} ; the output from **vertical_filter** is the data from **horizontal_filter** for parameters in *PV*. Figure 7 uses data from the examples in Figs. 5 and 6 to show how the **vertical_filter** step works.

The chain comprising the partitions generation and the horizontal and vertical filters is crucial to minimize the *Cold Start* problem (Lika, Kolomvatsos & Hadjiefthymiades, 2014). *Cold Start* is caused by the lack of ideal operating conditions for an algorithm, specifically in the recommender systems. This problem occurs, for example, when there are few users for the neighborhood definition with a similar user profile or lack of ratings for enough items. *FReeP* can also be affected by *Cold Start* problem. If only all preferences were used at one time for partitioning the provenance data, in some cases, it could be observed that the resulting partition would be empty. This is because there could be an absence of any of the user’s preferences in the provenance data. Therefore, generating multiple partitions with subsets of preferences decreases the chance of obtaining only empty partitions. However, in the worst case where none of the user’s preferences are present in the workflow provenance, *FReeP* will not perform properly, thus failing to make any recommendations.

After the partitions generation and horizontal and vertical filters are discovered, there is a filtered data set that follows part of the user’s preferences. These provenance data that will generate the Machine Learning model have numerical and categorical domain parameters. However, traditional Machine Learning models generally work with numerical data because the generation of these models, in most cases, involves many numerical calculations. Therefore, it is necessary to codify these categorical parameters to a numerical representation. The technique used here to encode categorical domain parameters to numerical representation is *One-Hot encoding* (Coates & Ng, 2011). This technique consists of creating a new binary attribute, that is, the domain of this new attribute is 0 or 1, for each different attribute value present in dataset.

The encoded provenance data allows building Machine Learning models to make predictions for the target parameter under the step **hypothesis_generation**. The model generated has the parameter *y* as class variable, and the other parameters present in **vertical_filter** step output data are the attributes used to generalize the hypothesis. The model can be a classifier, where the model’s prediction is a single recommendation value, or a *ranker*, where its prediction is an ordered list of values, of the value most suitable for the recommendation to the least suitable.

With a model created, we can use it to recommend the value for the target parameter. This step is represented in FReeP as **recommended**, and the recommendation of parameter *y* is made from the user’s preferences. It is important to emphasize that the model’s training data may contain parameters that the user did not specify any preference. In this case, an attribute of the instance submitted for the hypothesis does not have a defined value. To clarify the problem, let *PW* be all workflow parameters set, *PP* the parameters of workflow for which preference values have been defined; *PA* the parameters present in the partition rules of an iteration over the partitioning rules; and *PV* = (*PW* − *PP*) ∪ (*PP* ∩ *PA*) ∪ {*y*}, there may be parameters *p* ∈ *PV* | *p ∉ PP*, and for those parameters *p* there are no values defined a priori. To handle this problem, the average values present in the provenance data are used to fill in the numerical attributes’ values and the most frequent values in the provenance date for the categorical attributes.

All predictions generated by **recommend** step, which is within the iteration over the partitioning rules, are stored. The last algorithm step, **elect_recommendation**, uses all of these predictions as votes to define which value should be recommended for the target parameter. When an algorithm instance is setup to return a *classifier* type model in **hypothesis_generation** step, the most voted value is elected as the recommendation. On the other hand, when an algorithm instance is setup to return a *ranker* type model in **hypothesis_generation** step, the strategy is *Borda Count*. The use of the *Borda Count* strategy seeks to take advantage of the list of lists form that the saved votes acquire when using the *ranker* model. This list of lists format occurs because the *ranker* prediction is a list, and since there are as many predictions as partitioning rules, the storage of these predictions takes the list of lists format.

### Discrete and continuous domain parameter value recommendation

The naive version of *FReeP* allowed evaluating the algorithm’s proposal. The proposal showed relevant results after initial tests (presented in next section), so efforts were focused on improving its performance and utility. In particular, the following problems have been identified: (1) User has some restriction to set his/her parameters preferences; (2) The categorical domain parameters when used as a class variable (parameters for recommendation) are treated as well as they are present in the input data; (3) Machine Learning models used can only learn when the class (parameter) variable has a discrete domain; (4) All partitions generated by workflow parameters *powerset* present in user preferences are used as partitioning rules for the algorithm.

Regarding problem 1, in Algorithm 1, the user was limited to define his preferences with the equality operator. Depending on the user’s preferences, the equality operator is not enough. With this in mind, the *Enhanced FReeP* allows for the user to have access to the relational operators: ==,>,>=,<,<= and != to define his/her preferences. In addition, two logical operators are also supported in setting preferences: | and &. Preferences with combination of supported operators is also allowed, for example: (*a* > 10) | (*at* < 5).

**Algorithm 1:** Naive FReeP-Discrete.

**Require:**
}{}y:{\rm recommendation\; target \;parameter}
}{}P:\{ (param,val)|param{\rm \;is\; a\; workflow\; parameter,\; valis\; the\; preference\; value\; for\; }param\}
}{}D:\{ \{ (param_1^1,val_1^1),\ldots,(param_1^l,val_1^l),\ldots(param_l^m,val_l^m)\} |l{\rm \;is\; the\; workflow\; parameters\; number},\; m{\rm \;is\; the\; provenance\; dataset\; length\} }
1: **procedure** FReeP(y, P, D)
2: *partitions* ← *partitions*_*generation*(*P*, *D*)
3: *votes* ← ø
4: **for each** *partition* ∈ *partitions* **do**
5: *data* ← *horizontal*_*filter*(*D*, *partition*)
6: *data* ← *vertical*_*filter*(*data*, *partition*)
7: *data* ← ′ *preprocessing*(*data*)
8: *model* ← *hypothesis*_*generation*(*data*′, *y*)
9: *vote* ← *recommend*(*model*, *y*)
10: *votes* ← *votes* ∪ {*vote*}
11: *recommendation* ← *elect*_*recommendation*(*votes*)
12: **return** *recommendation*

However, by allowing users to define their preferences in this way we create a problem when setting up the instances for **recommendation** step. As seen, *PW* represents all workflow parameters set, *PP* are workflow parameters that preference values have been set; *PA* the parameters present in the partitioning rules of an iteration over the partition rules; and *PV* = (*PW* − *PP*) ∪(*PP* ∩ *PA*) ∪ {*y*}. Thus, there may be parameters *p* ∈ *PV* | *p ∉ PP*, and for those parameters *p*, there are no values defined a priori. This enhanced version of the proposal allows the user’s preferences to be expressed in a more relaxed way, demanding to create the instances used in the step **recommendation** that include a range (or set of values). To handle this isse, all possible instances from preference values combinations were generated. In case the preference is related to a numerical domain parameter and is defined in terms of values range, like *a* ≤ 10.5, *FReeP* uses all values present in the source provenance database that follows the preference restriction. It is important to note that for both numerical and categorical parameters, the combination of possible values are those present in the provenance database and that respect the user’s preferences. Then, predictions are made for a set of instances using the model learned during the training phase.

Regarding problem 2, the provenance database, in general, present attributes with numerical and categorical domains. It is *FReeP* responsibility to convert categorical values into numerical representation due to restrictions related to the nature of the training algorithms of the Machine Learning models, e.g., *Support Vector Machines (SVM)* (Wang, 2005).

This pre-processing step was included in Algorithm 2 as **classes_preprocessing** step. The preprocessing consists in exchanging each distinct categorical value for a distinct integer. Note that the encoding of the parameter used as a class variable in the model generation is different from the encoding applied to the parameters used as attributes represented by the step **preprocessing**.

**Algorithm 2:** Enhanced FReeP.

**Require:**
}{}y:{\rm recommendation \;target\; parameter}
}{}P:\{ (param,val)|param{\rm \;is\; a\; workflow\; parameter,\; valis\; the\; preference\; value\; for\; }param\}
}{}D:\{ \{ (param_1^1,val_1^1),\ldots,(param_1^l,val_1^l),\ldots(param_l^m,val_l^m)\} |l{\rm \;is\; the\; workflow\; parameters\; number},\; m{\rm \;is\; the\; provenance\; dataset\; length\} }
1: **procedure** FReeP(y, P, D)
2: *D*′ ← *classes*_*preprocessing*(*D*)
3: *partitions* ← *optimized*_*partitions*_*generation*(*P*, *D*′)
4: *votes* ← ø
5: **for each** *partitionpartition* ∈ *partitions* **do**
6*: data* ← *horizontal*_*filter*(*D*′, *partition*)
7: *data* ← *vertical*_*filter*(*data*, *partition*)
8: *model*_*type* ←*model*_*select*(*data*, *y*)
9: *data*′ ← *preprocessing*(*data*)
10: *model* ← *hypothesis*_*generation*(*data*′, *y*, *model*_*type*)
11: *vote* ← *recommend*(*model*, *y*)
12: *votes* ← *votes* ∪ {*vote* }
*13: recommendation* ← *elect*_*recommendation*(*votes*)
14: **return** *recommendation*

Concerning problem 3, by using classifiers to handle a continuous domain class variable degrades the performance results. Performance degradation happens because the numerical class variables are considered as categorical. For continuous numerical domain class variables, the Machine Learning models suggested are *Regressors* (Myers & Myers, 1990). In this way, the *Enhanced FReeP* checks the parameter *y* domain, which is the recommendation target parameter, represented as **model_select** step in Algorithm 2.

To analyze problem 4, it is important to note that after converting categorical attributes *One-Hot encoding* in **preprocessing** step, the provenance database will have a considerable increase in the number of attributes. Also, after categorical attributes encoding in **preprocessing** step, the parameters extracted from the user’s preferences, are also encoded for **partitions_generation** step. In Algorithm 1, the partitioning rules *powerset* is calculated on all attributes derived from the original parameters after *One-Hot encoding*. If FReeP uses the *powerset* generated from the parameters present in the user’s preferences set as partitioning rules (in the **partitions_generation** step), it can be very costly. Thus, using the *powerset* makes the complexity of the algorithm becomes exponential according to the parameters present in the user’s preferences set. Alternatives to select the best partitioning rules and handle the exponential cost are represented in Algorithm 2 as **optimized_partitions_generation** step. The two strategies proposed here are based on *Principal Components Analysis (PCA)* (Garthwaite et al., 2002) and the *Analysis of variance (ANOVA)* (Girden, 1992) statistical metric.

The strategy based on *PCA* consists of extracting *x* principal components from all provenance database, *pca*_*D*_, and for each *pt* ∈ *partitions*, }{}pca_{pt}^i, which are *pt* partition principal components. Then, the norms are calculated ||pca_D_ − }{}pca_{pt}^i||, and from that *n* partitioning rules are selected that generated }{}pca_{pt}^i such that ||pca_D_ − }{}pca_{pt}^i|| resulted in the lowest calculated values. Note that both *x* and *n* are defined parameters when executing the algorithm. In summary, the *PCA* strategy will select the partitions where the main components extracted are the closest to the principal components of the original provenance dataset.

*ANOVA* strategy seeks the *n* partitioning rules that best represent *D*, selecting those that generate partitions where the data variance is closest to *D* data variance. In short, original data variance and data variance for each partition are calculated using the *ANOVA* metric, then partitions with most similar variance to the original provenance data are selected. Here, the *n* rules are defined in terms of the data percentage required to represent the entire data set, and that parameter must also be defined in algorithm execution. Using *PCA* or *ANOVA* partitioning strategies means that the partitioning rules used by FReeP can be reduced, depending on the associated parameters that need to be defined.

### Recommendation for *n* Parameters at a time

Algorithms 1 and 2 aim at producing single parameter recommendation at a time. However, in a real usage scenario of scientific workflows, the WfMS will probably need to recommend more than one parameter at a time. A naive alternative to handle this problem is to execute Algorithm 2 for each of the target parameters, always adding the last recommendation to the user’s preference set. This alternative assumes that the parameters to be recommended are independent random variables. One way to implement this strategy is by using a *classifiers chain* (Read et al., 2011).

Nevertheless, this naive approach neglects that the order in which the target parameters are used during algorithm interactions can influence the produced recommendations. The influence is due to parameter dependencies that can be found between two (or more) workflow activities (e.g., two activities consume a parameter produced by a third activity of the workflow). In Fig. 3, the circles represent the activities of workflow, so activities 2 and 3 are preceded by activity 1 (e.g., they consume the output of activity 1). Using this example, we can see that it is possible that there is a dependency relationship between the parameters *param2* and *param3* with the parameter *param1*. In this case, the values of *param2* and *param3* parameters can be influenced by parameter *param1* value.

In order to deal with this problem, FReeP leverages the *Classifiers Chains Set* (Read et al., 2011) concept. This technique allows for estimating the joint probability distribution of random variables based on a *Classifiers Chains Set*. In this case, the random variables are the parameters for which values are to be recommended, and the joint probability distribution concerns the possible dependencies between these parameters. The *Classifiers Chains* and *Classifiers Chains Set* are techniques from *Multi-label Classification* (Tsoumakas & Katakis, 2007) Machine Learning task.

**Figure 8:**
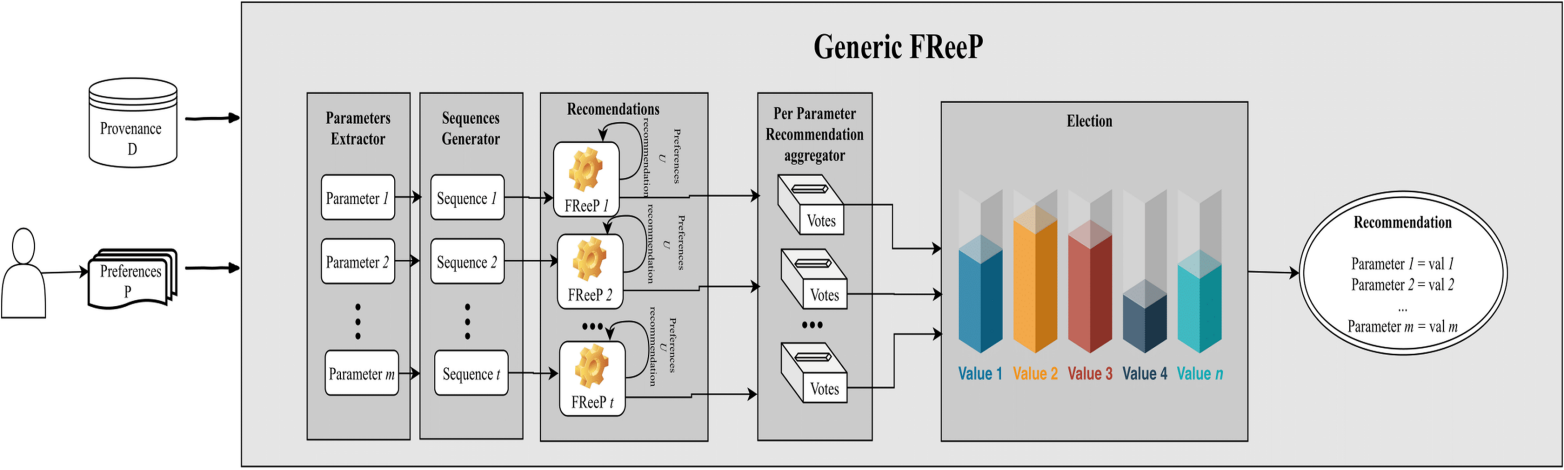
*Generic FReeP* architecture overview.

Figure 8 depicts an architecture overview for the proposed algorithm named as *Generic FReeP* that recommends *n* parameters simultaneously. The architecture presented in Fig. 8 shows that the solution developed to make *n* parameter recommendations at a time is a packaging of *FReeP* algorithm to one parameter. This final approach is divided into five steps: identification of parameters for the recommendation, generation of ordered sequences of these parameters, iteration over each of the sequences generated with the addition of each recommendation from *FReeP* to the user preferences set, separation of recommendations by parameter and finally the choice of value recommendation for each target parameters. The formalization can be seen in Algorithm 3.

**Algorithm 3:** Generic FReeP.

**Require:**
}{}P:{\rm recommendation \;target\; parameter}
}{}D:\{ (param,val)|param{\rm \;is\; a\; workflow\; parameter,\; valis\; the\; preference\; value\; for\; }param\}
}{}D:\{ \{ (param_1^1,val_1^1),\ldots,(param_1^l,val_1^l),\ldots(param_l^m,val_l^m)\} |l{\rm \;is\; the\; workflow\; parameters\; number},\; m{\rm \;is\; the\; provenance\; dataset\; length\} }
N :number of random sequences orders to be generated
1: **procedure** Generic FReeP(P, D, N)
2: *target*_*parameters* ← *parameters*_*extractor*(*P*, *D*)
3: *votes* ← ø
4: **for each** *param* ∈ *target*_*parameters* **do**
5: *votes* ← *votes* ∪ {(*param*, []) }
6: *ordered*_*sequences* ← *sequence*_*generator*(*target*_*parameters*, *N*)
7: **for each** *sequence* ∈ *ordered*_*sequences* **do**
8: *preferences*_*tmp* ← *P*
9: **for each** *param* ∈ *sequence* **do**
10: *recommendation* ← *FReeP*(*param*, *preferences*_*tmp*, *D*)
11: *votes*[*param*] ← *votes*[*param*] ∪ *recommendation*
12: *new*_*preference* ← *generate*_*preference*(*param*, *recommendation*)
13: *preferences*_*tmp* ← *preferences*_*tmp* ∪ *new*_*preference*
14: *response* ← ø
15: **for each** (*param*, *values*) ∈ *votes* **do**
16: *response*[*param*] *most*_*voted*(*values*)
17: **return** *response*

The first step **parameters_extractor** extracts the workflow parameters that are not present in the users’ preferences and will be the targets of the recommendations. Thus, all other parameters that are not in the user’s preferences will have recommendation values.

Lines 4 and 5 of the algorithm comprise the initialization of the variable responsible for storing the different recommendations for each parameter during the algorithm execution. Then, the list of all parameters that will be recommended is used for generating different ordering of these parameters, indicated by **sequence_generators** step. For example, let *w* be a workflow with 4 *p* parameters and let *u* be an user with *pr*_1_ and *pr*_3_ preferences for the *p*_1_ and *p*_3_ parameters respectively. The parameters to be recommended are *p*_2_ and *p*_4_, in this case two possible orderings are: {*p*_2_, *p*_4_} and {*p*_4_, *p*_2_}. Note that the number of sorts used in the algorithm are not all possible sorts, in fact *N* of the possible sorts are selected at random.

Then, the algorithm initializes an iteration over each of the sorts generated by the step **sequence_generators**. Another nested iteration over each parameter present in the current order also begins. An intuitive explanation of the algorithm between lines 9 and 13 is that each current sequence parameter is used together with the user’s preferences for its recommendation. At the end of the recommendation of one of the ordering parameters, the recommendation is incorporated into the preferences set used in the recommendation of the next ordering parameter. In this iteration, the recommendations are grouped by parameter to facilitate the election of the recommended value for each target parameter.

The step of iterating over the generated sequences, always adding the last recommendation to the set of preferences, is the *Classifiers Chains* concept. To deal with the dependency between the workflow parameters that can influence a parameter value recommendation, the step that generates multiple sequences of parameters, combined with the *Classifiers Chains*, is the *Classifiers Chains Set* concept.

Finally, to choose the recommendation for each target parameter, a vote is taken on lines 15 and 16. The **most_voted** procedure makes the majority election that defines the target parameter recommendation value. This section presented three algorithms that are part of the FReep approach developed for the parameter recommendation problem in workflows. The proposals covered two main scenarios for parameters value recommendation (single and multiple parameter at a time).

## Experimental Evaluation

This section presents the experimental evaluation of all versions of FReeP. First, we present the workflows used as case studies namely SciPhy (Ocaña et al., 2011) and Montage (Jacob et al., 2009). Following we present the experimental and environment setups. Finally, we discuss the results.

### Case studies

**Figure 9:**
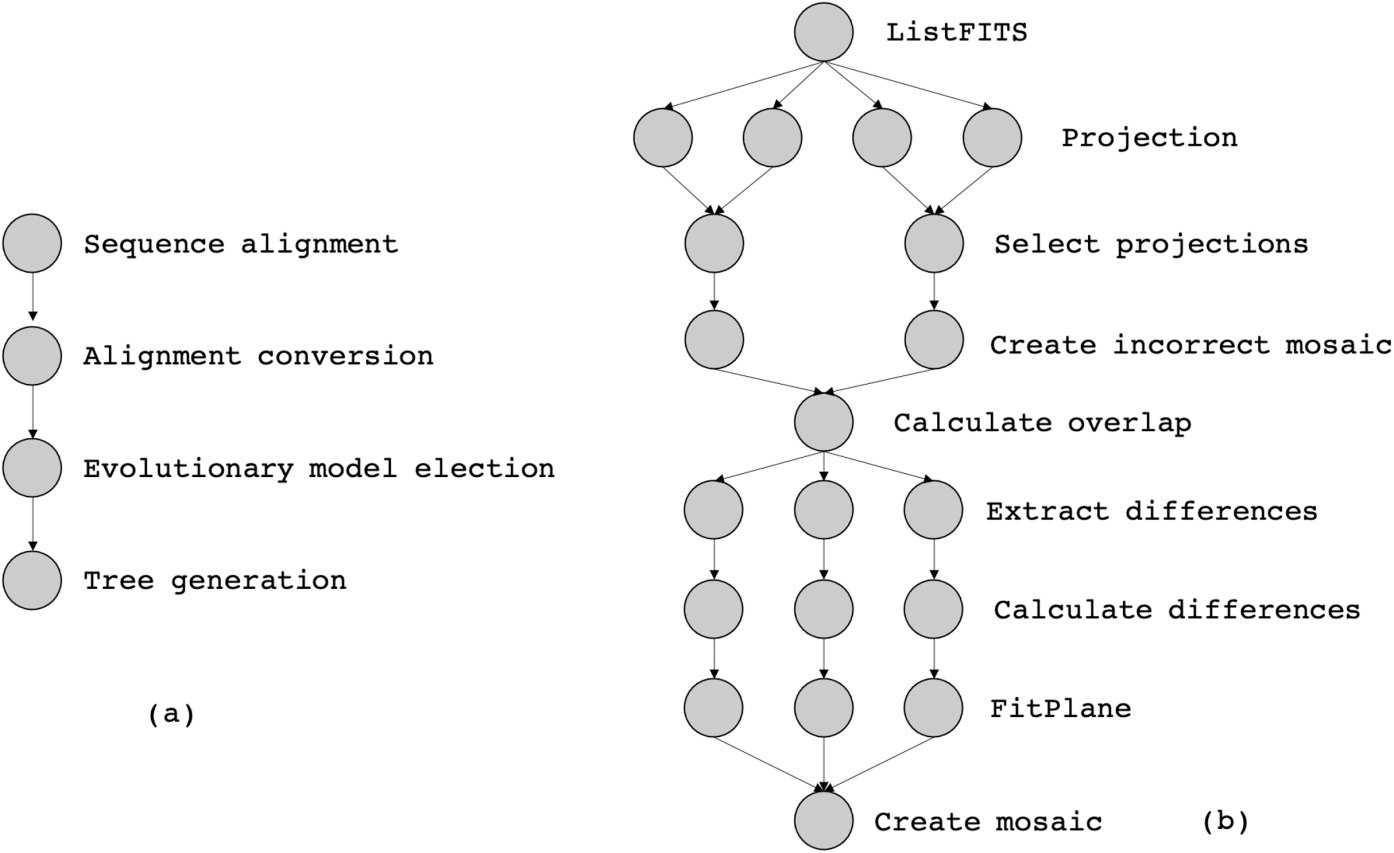
The abstract specification of (a) SciPhy and (b) Montage.

In this article, we consider two workflows from bioinformatics and astronomy domains, namely SciPhy (Ocaña et al., 2011) and Montage (Jacob et al., 2009), respectively. SciPhy is a phylogenetic analysis workflow that generates phylogenetic trees (a tree-based representation of the evolutionary relationships among organisms) from input DNA, RNA and aminoacid sequences. SciPhy has four major activities as presented in Fig. 9A: (i) sequence alignment, (ii) alignment conversion, (iii) evolutionary model election and (iv) tree generation. SciPhy has been used in scientific gateways such as BioInfoPortal (Ocaña et al., 2020). SciPhy is a CPU-intensive workflow, bacause many of its activities (especially the evolutionary model election) commonly execute for several hours depending on the input data and the chosen execution environment.

Montage (Jacob et al., 2009) is a well-known astronomy workflow that assembles astronomical images into mosaics by using FITS (Flexible Image Transport System) files. Those files include a coordinate system and the image size, rotation, and WCS (World Coordinate System) map projection. Figure 9B shows the *montage* activities: *(i)* ListFITS, which extracts compressed FITS files, *(ii)* Projection, which maps the astronomical positions into a Euclidean plane, *(iii)* SelectProjections, which joins the planes into a single mosaic file, and *(iv)* CreateIncorrectedMosaic, which creates an overlapping mosaic as an image. Programs *(v)* CalculateOverlap, *(vi)* ExtractDifferences, *(vii)* CalculateDifferences, *(viii)* FitPlane, and *(ix)* CreateMosaic refine the image into the final mosaic. Montage is a data-intensive workflow, since one single execution of Montage can produce several GBs of data.

### Experimental and environment setup

All *FReeP* algorithms presented in this article were implemented using the *Python* programming language. *FReeP* implementation also benefits from *Scikit-Learn* (Pedregosa et al., 2011) to learn and evaluate the Machine Learning models, *numpy* (Van der Walt, Colbert & Varoquaux, 2011), a numerical data manipulation library; and *pandas* (McKinney, 2011), which provides tabular data functionalities.

The machine specification where experiments were performed is a *CPU Celeron (R) Dual-Core T3300 @ 2.00 GHz × 2* processor, *4GB DDR2* RAM and *132 GB* HDD. To measure recommendations performance when the parameter is categorical, *precision* and *recall* are used as metrics. *Precision* and *recall* are metrics widely used for the quantitative assessment of recommender systems (Herlocker et al., 2004; Schein et al., 2002). Eq. (1) defines *precision* and Eq. (2) defines *recall*, following the recommender vocabulary, where *TR* is the correct recommendation set and *R* is all recommendations set. An intuitive explanation to *precision* is that it represents the most appropriate recommendations fraction. Still, *recall* represents the appropriate recommendation fraction that was made.

(1) }{}precision = \displaystyle{{{\rm \parallel }TR \cap R{\rm \parallel }} \over {{\rm \parallel }R{\rm \parallel }}}

(2) }{}recall = \displaystyle{{{\rm \parallel }TR \cap R{\rm \parallel }} \over {{\rm \parallel }TR{\rm \parallel }}}

(3) }{}MSE = \displaystyle{1 \over n}\sum\limits_{i = 1}^n {(RV - TV)^2}

When the parameter to be recommended is numerical, the performance of FReeP is evaluated with *Mean Square Error (MSE)*. The MSE formula is given by Eq. (3) where *n* is the recommendations number, *TV* is the correct recommendation values set, and *RV* is the recommended values set.

## Dataset

The datasets used are provenance data extracted from real executions of the workflows *SciPhy* (all executions) using *SciCumulus* Workflow Management System and *Montage* (part of the executions with SciCumulus) and part of the execution data gathered at the Workflow Generator site (https://confluence.pegasus.isi.edu/display/pegasus/WorkflowGenerator). This site provides instances of real workflow for evaluation of algorithms and systems on a range of workflow sizes. All data within these workflow traces is gathered from real executions of scientific workflows on the grid and in the cloud from the Pegasus’ team in ISI at the University of Southern California. The SciPhy executions consumed from 200 up to 500 fasta files downloaded from RefSeq database. The Montage executions consumed from 50 up to 100 FIT files obtained from the “Two Micron All-Sky Survey”^1^ 1Data sources are available at http://irsa.ipac.caltech.edu.. In the case of SciPhy, the executions were performed by 3 different users (one expert and 2 undergraduate students). In the case of Montage (the executions in SciCumulus were performed by an undergraduate student and the ones downloaded from the Workflow Generator site were performed by experts).

Table 1 summarizes the main characteristics of the datasets. The *Total Records* column shows the number of past executions of each workflow. Each dataset record can be used as an example for generating Machine Learning models during the algorithm’s execution. As seen, the *SciPhy* dataset is relatively small compared to *Montage*. The column *Total Attributes* shows how many activity parameters are considered in each workflow execution. Both *workflows* have the same number of categorical domain parameters, as presented in the column *Categorical Attributes*. *Montage* has more numeric domain parameters than *SciPhy*, as shown in the *Numerical Attributes* column.

**Table 1:** Dataset characteristics.

Dataset	Total records	Total attributes	Categorical attributes	Numerical attributes
*Sciphy*	376	6	2	4
*Montage*	1,565	8	2	6

Statistics on the *SciPhy* numerical attributes are shown in Table 2. This table presents the minimum and maximum values of each attribute, in addition to the standard deviation. The attribute *prob1* (probability of a given evolutive relationship is valid) has the highest standard deviation, and its range of values is the largest among all attributes. The *prob2* attribute (probability of a given evolutive relationship is valid) has both a range of values and the standard deviation similar to *prob1*. The standard deviation of the values of *num_aligns* (total number of alignments in a given data file) is very small, while the attribute *length* (maximum sequence length in a specific data file) has a high standard deviation, considering its values range.

**Table 2:** *SciPhy* dataset statistics.

Parameter	Minimum value	Maximum value	Standard deviation
num_aligns	9.00	11.00	0.21
length	85.00	1,039.00	169.90
prob1	634.67	5,753.52	1,103.43
prob2	635.87	5,795.28	1,101.76

The *Montage* numerical attributes, shown in Table 3, in most of the cases, have smaller standard deviation than the *SciPhy*. On average, *Montage* attributes also have a smaller values range than *SciPhy* dataset attributes. Also, in *Montage* dataset, the *crota2* attribute (a float value that represents an image rotation on sky) has the largest values range and the largest standard deviation. The *dec* (an optional float value that represents Dec for region statistics) and *crval2* (a float value that represents Axis 2 sky reference value in Montage workflow) attributes have close statistics and are the attributes with the smallest data range and the smallest *Montage* data standard deviation.

**Table 3:** *Montage* dataset statistics.

Parameter	Minimum value	Maximum value	Standard deviation
cntr	0.00	134.00	35.34
ra	83.12	323.90	91.13
dec	−27.17	28.85	17.90
crval1	83.12	323.90	91.13
crval2	−27.17	28.85	17.90
crota2	0.00	360.00	178.64

**Figure 10:**
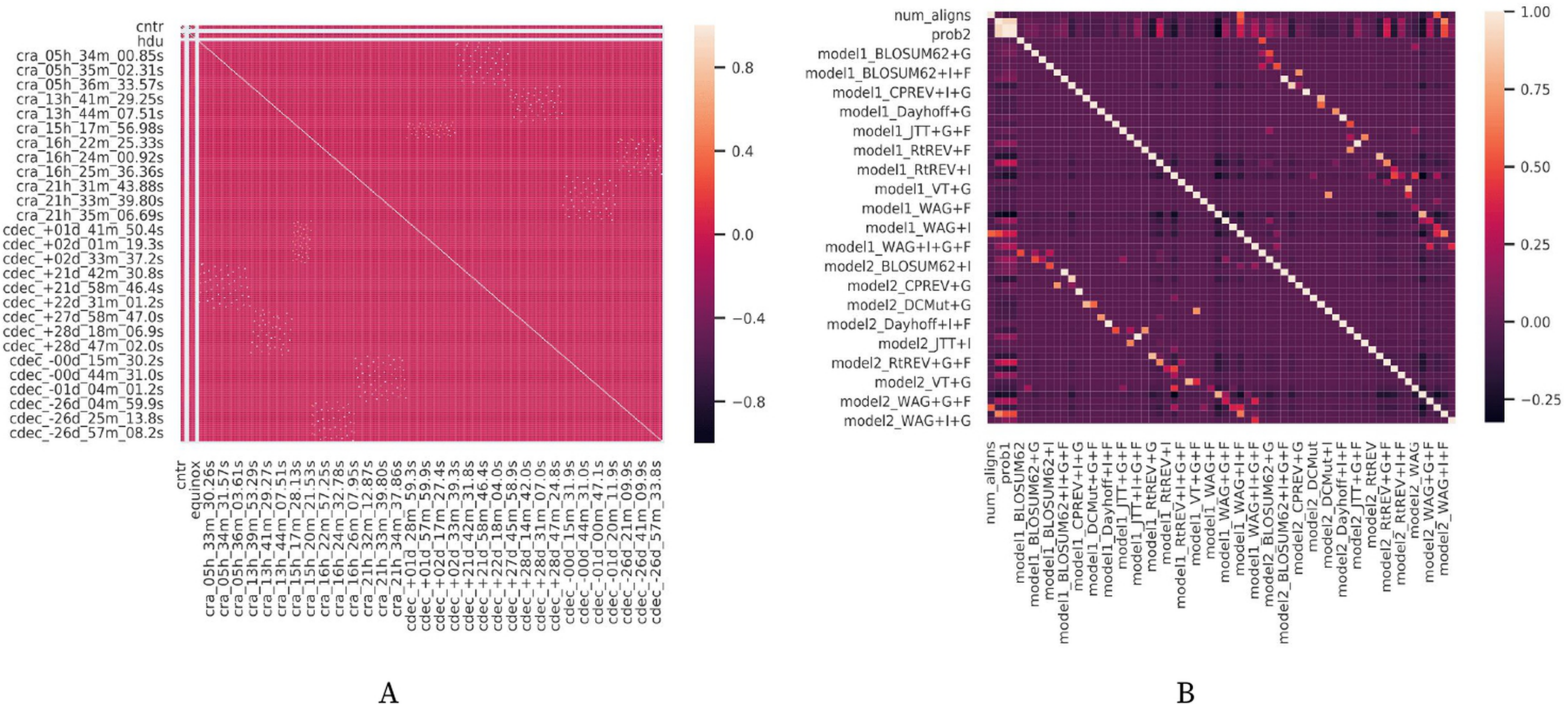
Datasets attributes correlation matrices.

In Fig. 10, it is possible to check the correlation between the different attributes in the datasets. It is notable in both Figs. 10A and 10B that the attributes (i.e., workflow parameters) present a weak correlation. All those statistics are relevant to understand the results obtained by the experiments performed from each version of FReeP algorithm.

### Discrete domain recommendation evaluation

This experiment was modeled to evaluate *FReeP*’s algorithm key concepts using the naive version presented in Algorithm 1, that was developed to recommend one discrete domain parameter at a time. This experiment aims at evaluating and comparing the performance of *FReeP* when its *hypothesis_generation* step instantiates either a single *classifier* or a *ranker*. The *ranker* tested as a model was implemented using the *Pairwise Label Ranking* technique. *K Nearest Neighbors* (Keller, Gray & Givens, 1985) classifier is used as the classifier of this *ranker* implementation. The *k* parameter of *K Nearest Neighbors* classifier was set as 3, 5, 7 for both the *ranker* and *classifier*. The choice of *k* ∈ {3,5,7} is because small datasets are used, and thus *k* values greater than 7 do not return any neighbors in the experiments.

### Experiment 1. Algorithm 1 evaluation script

1. The algorithm is instantiated with the *classifier* or *ranker* and a recommendation target workflow parameter.
2. The provenance database is divided into *k* parts to follow a *K-Fold Cross Validation* procedure (Kohavi, 1995). At each step, the procedure takes *k* − 1 parts to train the model and the 1 remaining part to make the predictions. In this experiment, *k* = 5.
3. Each workflow parameter is used as recommendation target parameter.
4. Each provenance record in test data is used to retrieve target parameter real value.
5. Parameters that are not the recommendation target are used as preferences, with values from current test record.
6. Then, algorithm performs recommendation and both the result and the value present in the test record for the recommendation target parameter are stored.
7. *Precision* and *recall* values are calculated based on all *K-Fold Cross Validation* iterations.

#### Results

**Figure 11:**
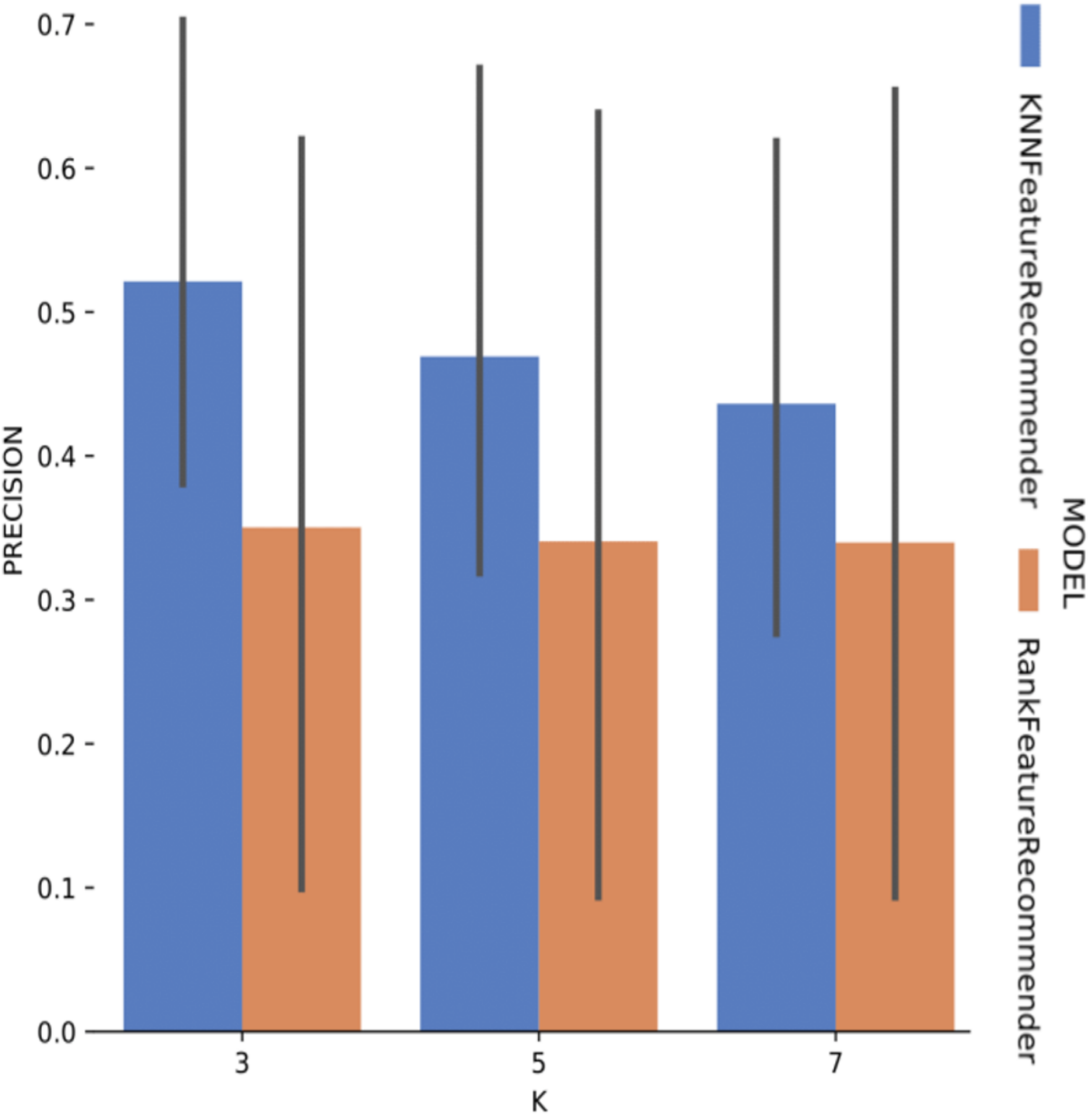
Precision results with *SciPhy* data.

**Figure 12:**
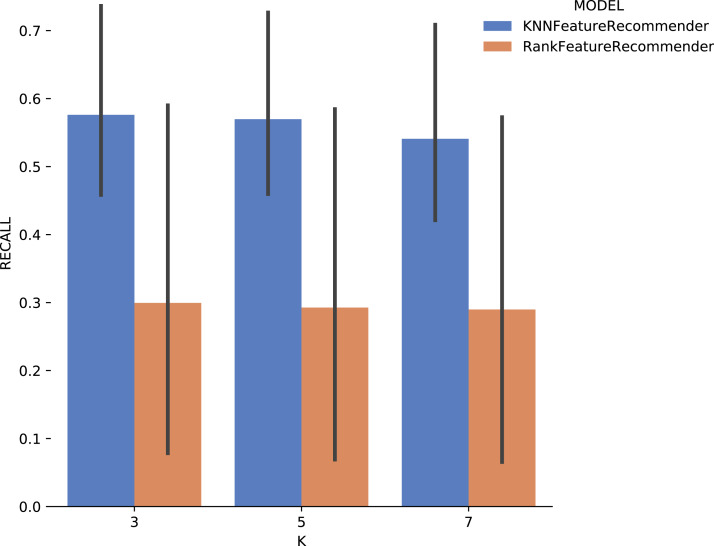
Recall results with *SciPhy* data.

Experiment 1 results are presented and analyzed based on the values of *precision* and *recall*, in addition to the execution time. Figure 11 shows that Algorithm 1 execution with *Sciphy* provenance database, using both the *classifier* and the *ranker*. Only KNN classifier with *k* = 3 gives a precision greater than 50%. Also, a high standard deviation is noticed. Even with unsatisfactory performance, Fig. 12 shows that *KNN* classifier presented better *recall* results than those for *precision*, both in absolute values terms and standard deviation, which had a slight decrease. In contrast, the *ranker recall* was even worse with the *precision* results and still present a very high standard deviation.

**Figure 13:**
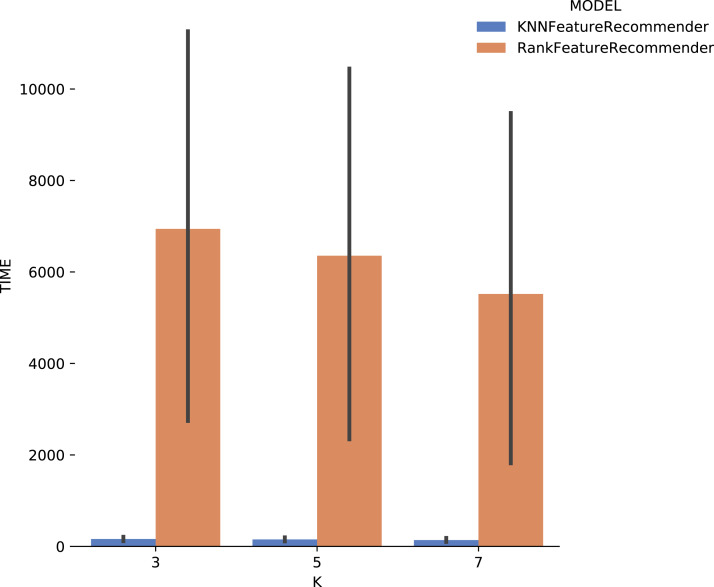
Experiment recommendation execution time with *SciPhy* data.

Figure 13 shows the execution time, in seconds, to obtain the experiment’s recommendations for SciPhy. The execution time of *ranker* is much more significant when compared to the time spent by the *classifier*. This behavior can be explained by the fact that the technique used to generate the *ranker* creates multiple *binary classifiers*. Another point to note is that the execution time standard deviation from *ranker* is also very high. It is important to note that when *FReeP* uses KNN, it is memory-based, since each recommendation needs to be loaded into main memory.

**Figure 14:**
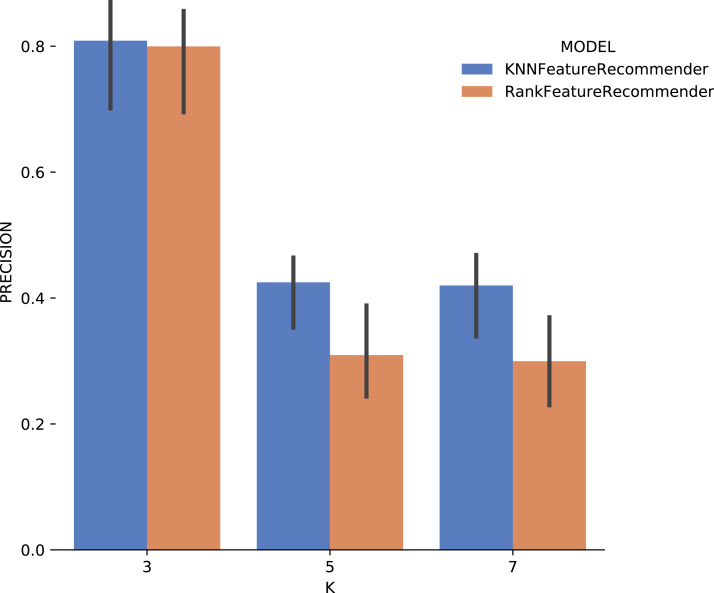
Precision results with *Montage* data.

Analyzing Fig. 14 (Montage) one can conclude that with the use of *k* = 3 for the classifier and for the *ranker* produces relevant results. The *precision* for this case reached 80%, and the standard deviation was considerably smaller compared to the *precision* results with *Sciphy* dataset in Fig. 11. For *k* ∈ {5,7}, the same results behavior was observed, considerably below those expected.

**Figure 15:**
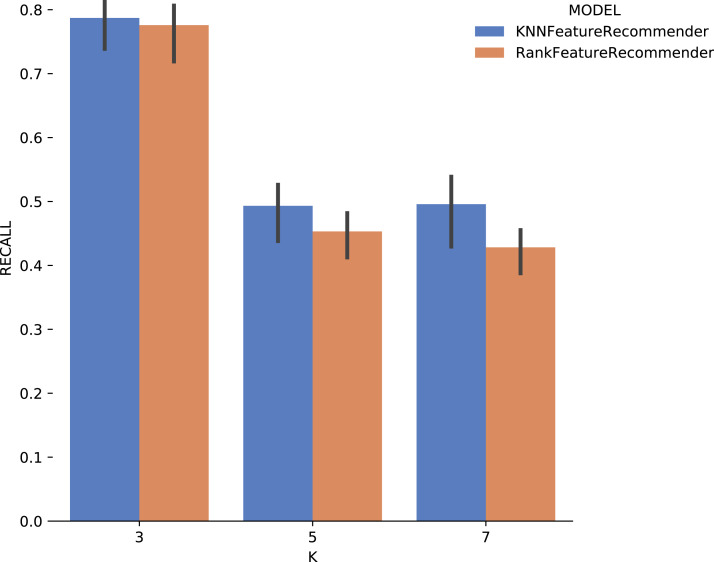
Recall results with *Montage* data.

**Figure 16:**
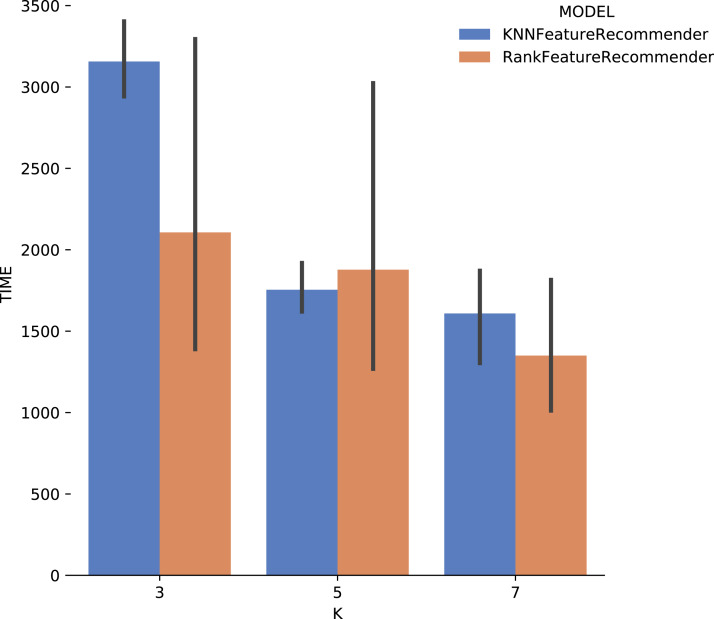
Experiment recommendation execution time with *Montage* data.

Considering the precision, Fig. 15 shows that the results for *k* = 3 were the best for both the classifier and for *ranker*, although for this case they did not reach 80% (although it is close). It can be noted that the standard deviation was smaller when compared to the standard deviations found for *precision*. One interesting point about the execution time of the experiment with *Montage* presented in Fig. 16 is that for *k* ∈ {3,7} the *ranker* spent less time than the *classifier*. This behavior can be explained because the *ranker*, despite being generated by a process where several classifiers are built, relies on binary classifiers. When used alone, the classifier needs to handle all class variables values, in this case, parameter recommendation values, at once. However, it is also important to note that the standard deviation for *ranker* is much higher than for the *classifier*.

In general, it was possible to notice that the use of *ranker* did not bring encouraging results. In all cases, *ranker precision* and *recall* were lower than those presented by the *classifier*. Besides, the standard deviation of *ranker* in the execution time spent results was also very high. Another point to be noted is that the best *precision* and *recall* results were obtained with the data from *Montage workflow*. These results may be linked to the fact that the *Montage* dataset has more records than the *Sciphy* dataset.

### Discrete and continuous domain recommendation evaluation

Experiment 1 was modified to evaluate the Algorithm 2 performance, yielding Experiment 2. Algorithm 2 was executed with variations in the choice of classifiers and regressors, partitions strategies, and records percentage from provenance database. All values per algorithm parameter are presented in Table 4.

**Table 4:** Algorithm 2 values per parameter used in Experiment 2.

Classifiers	Regressors	Partition strategy	Percentage
KNN	Linear Regression	PCA	30
SVM	KNR	ANOVA	50
Multi-Layer Perceptron	SVR		70
	Multi-Layer Perceptron		

### Experiment 2. Algorithm 2 evaluation script

1. Algorithm 2 is instantiated with a *classifier* or *regressor*, a partitioning strategy, percentage data to be returned by partitioning strategy, and a target workflow parameter.
2. Provenance database is divided using *K-Fold Cross Validation*, *k* = 5
3. Each provenance record on test data is used to retrieve the target parameter’s real value.
4. A random number *x* between 2 and parameters number present in provenance database is chosen to simulated preference number used in recommending target parameter.
5. *x* parameters are chosen from the remaining test record to be used as preferences.
6. Algorithm performs recommendation, and both result and test record value for the target parameter are stored.
7. Precision and recall, or *MSE* values are calculated based on all K-Fold Cross Validation iterations.

#### Results

Experiment 2 results are presented using *precision*, *recall*, and execution time for categorical domain parameters recommendations, while numerical domain parameters recommendations are evaluated using *MSE* and the execution time. Based on the results obtained in Experiment 1, only classifiers were used as Machine Learning models in Experiment 2, i.e., we do not consider rankers.

**Figure 17:**
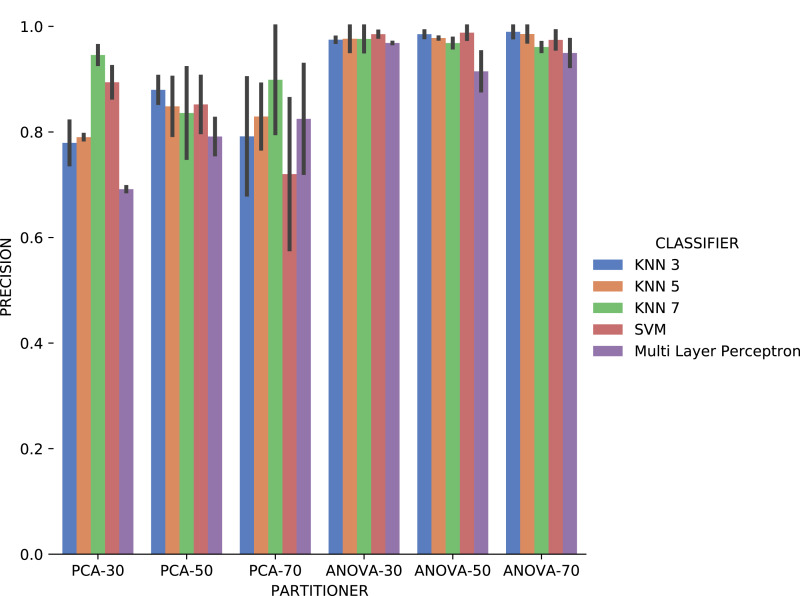
Precision results with *Sciphy* data.

The first observation when analyzing the *precision* data in Fig. 17 is that *ANOVA* partitioning strategy obtained better results than *PCA*. *ANOVA* partitioning strategy *precision* in absolute values is generally more significant, and variation in *precision* for each attribute considered for recommendation is lower than *PCA* strategy. The *classifiers* have very similar performance for all percentages of partitions in the *ANOVA* strategy. On the other hand, the variation in the percentages of elements per partition also reflects a more significant variation in results between the different classifiers. The *Multi Layer Perceptron (MLP)* classifier, which was trained using the *Stochastic Descending Gradient* (Bottou, 2010) with a single hidden layer, presents the worst results except in the setup that it follows the *PCA* partitioning strategy with a percentage of 70% elements in the partitioning. The *MLP* model performance degradation may be related to the fact that the numerical attributes are not normalized before algorithm execution.

**Figure 18:**
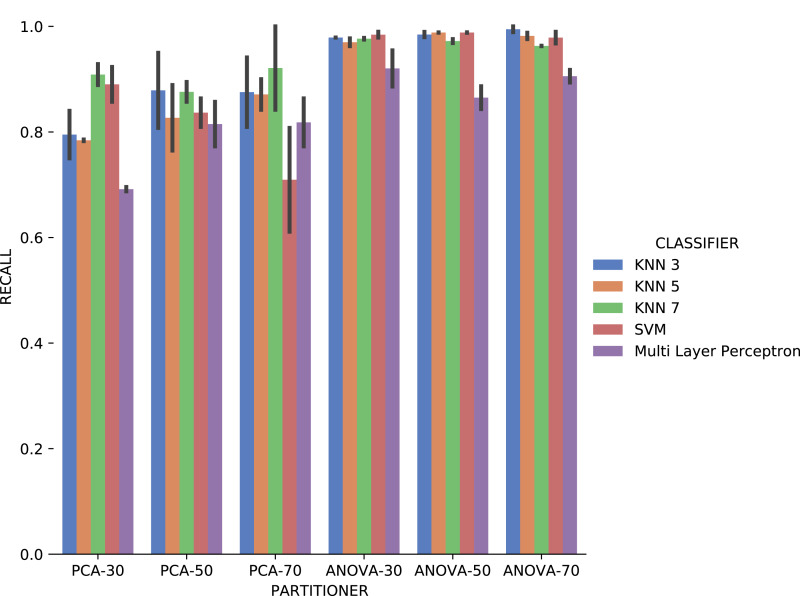
Recall results with *Sciphy* data.

*Recall* results, in Fig. 18 were very similar to *precision* results in absolute values. A difference is the smallest variation, in general, of *recall* results for each attribute used in the recommendation experiment. The *Multi Layer Perceptron* classifier presented a behavior similar to the *precision* results, with a degradation in the setup that includes *ANOVA* partitioning with 70% of the elements in the partitioning.

**Figure 19:**
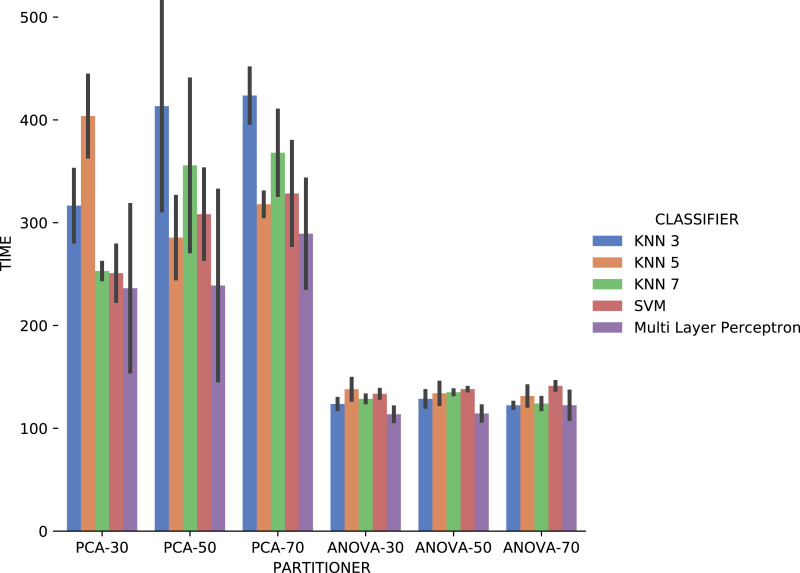
Experiment recommendation execution time with *Sciphy* data.

Figure 19 shows the average execution time in seconds during the experiment with categorical domain parameters in each setup used. Execution time of *ANOVA* partitioning strategy was, on average, half the time used with the *PCA* partitioning strategy. The execution time using different classifiers for each attribute is also much smaller and stable for *ANOVA* strategy than for *PCA*, regardless of element partition percentage.

Analyzing *precision*, *recall*, and execution time spent data jointly, *ANOVA* partitioning strategy showed the best recommendation performance for the categorical domain parameters of the *Sciphy* provenance database. Going further, the element partition percentage generated by the strategy has no significant impact on the results. Another interesting point is that a simpler classifier like *KNN* presented results very similar to those obtained by a more complex classifier like *SVM*.

**Figure 20:**
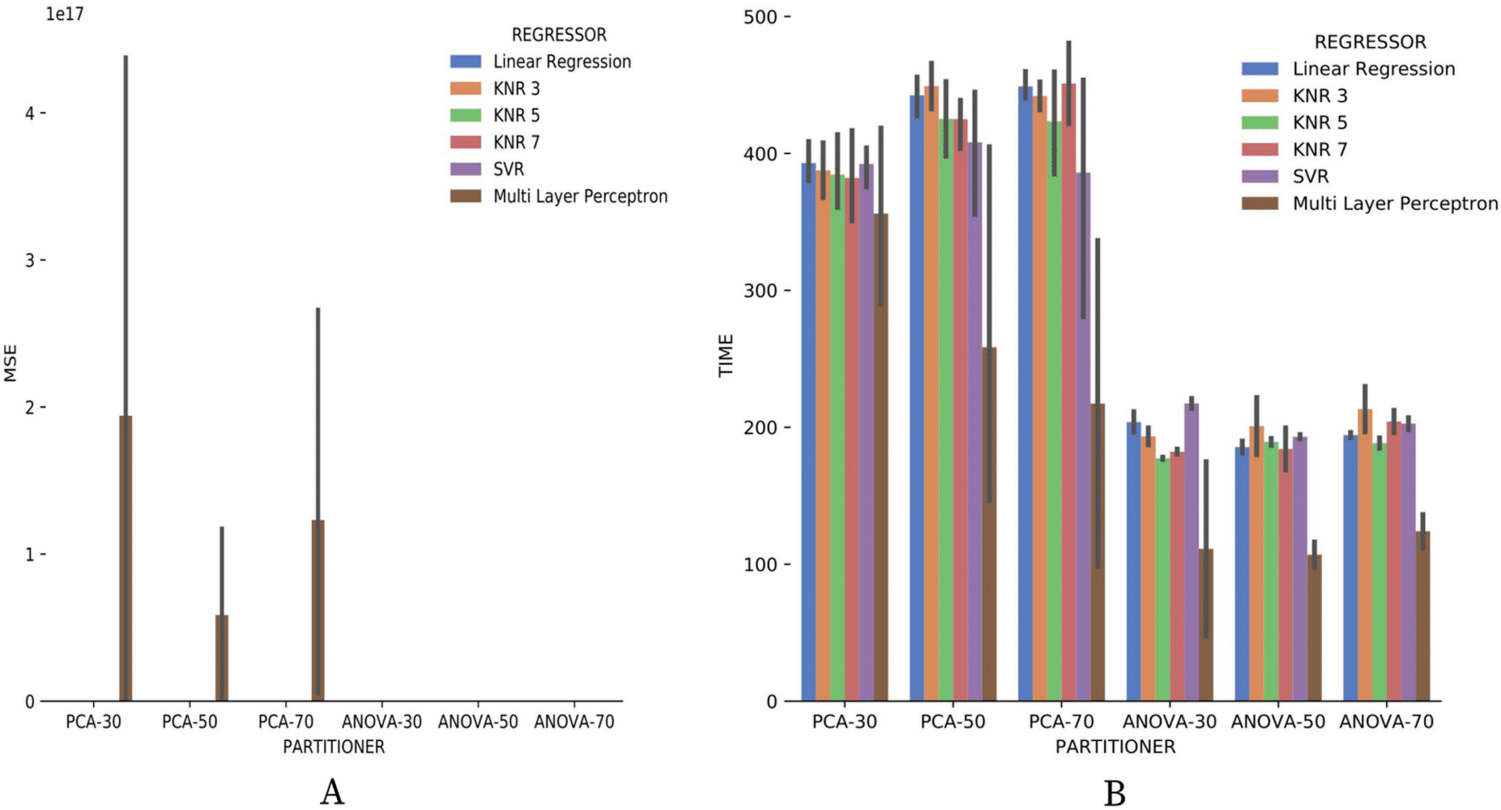
*MSE* results and recommendation execution time with *Sciphy* data.

Figure 20A brings the data from results obtained for the numerical domain parameter *Sciphy* provenance database. The data shows zero *MSE* in all cases, except for the use of *Multi Layer Perceptron* in the regression. This result can be explained by the small database and the few different values for each numerical domain parameter. Small values difference per parameter suggests that the *regressors* have no work to generate a result equal to what is already present in the database.

Looking at Fig. 20B, one can notice that, similar to the categorical domain parameters results, the execution time of *ANOVA* partitioning strategy is much less than the time used by the *PCA* strategy. Another similar point with categorical domain parameter results is the smaller and more stable *ANOVA* strategy results variation.

From all results obtained in the Experiment 2 using *Sciphy* provenance database, it can be noticed that the *ANOVA* partitioning strategy had the best performance. Further *precision*, *recall*, and *MSE* results, for the Algorithm 2 setup with *ANOVA* partitioning strategy also proved to be the one that performed the recommendations in the shortest time, generally in half the time that the *PCA* partitioning strategy. Note that the recommendation time can be treated as training time since the proposed algorithm has a memory-based approach. Finally, the choice of the generated partition size and the *classifier* or *regressor* used have no significant impact on the final result unless the *classifier* or *regressor* is based on *Multi Layer Perceptron* with the same parametrization used in this article.

**Figure 21:**
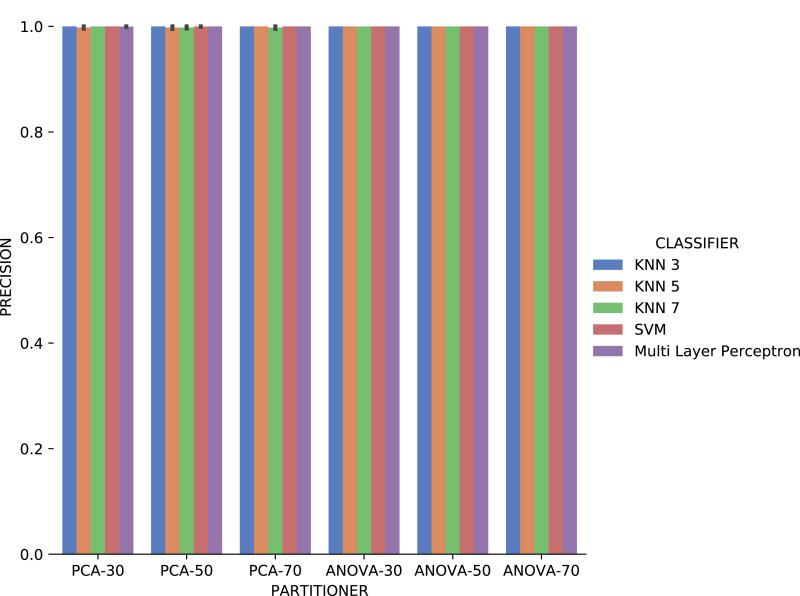
Precision results with *Montage* data.

**Figure 22:**
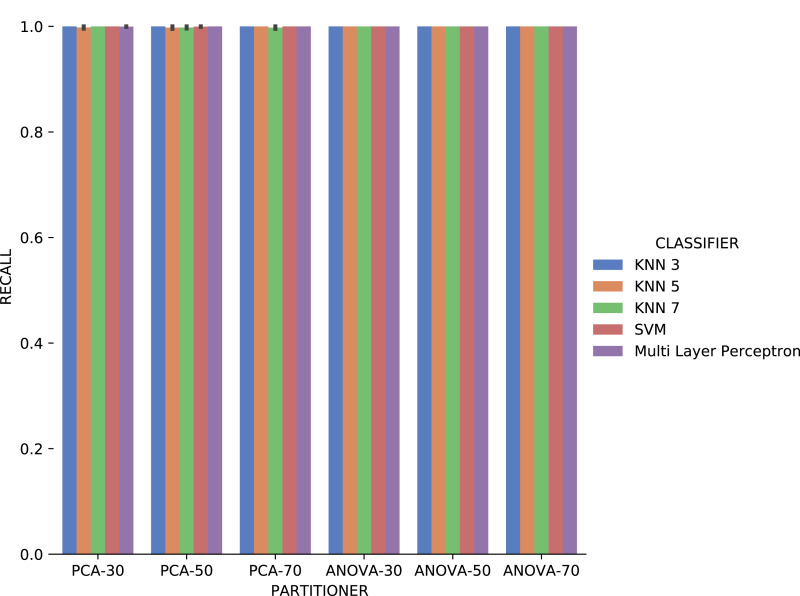
Recall results with *Montage* data.

Analyzing Fig. 21, *precision* results obtained with categorical domain parameters from *Montage* workflow provenance database is observed that in almost all the experiment setup variations evaluated, maximum performance is reached. As seen in Table 1, the *Montage* workflow provenance database used in the experiments has only two categorical domain parameters. The small variation in possible values in the database is an explanation for the *precision* results. The *recall* results in Fig. 22 are similar to the *precision* ones.

**Figure 23:**
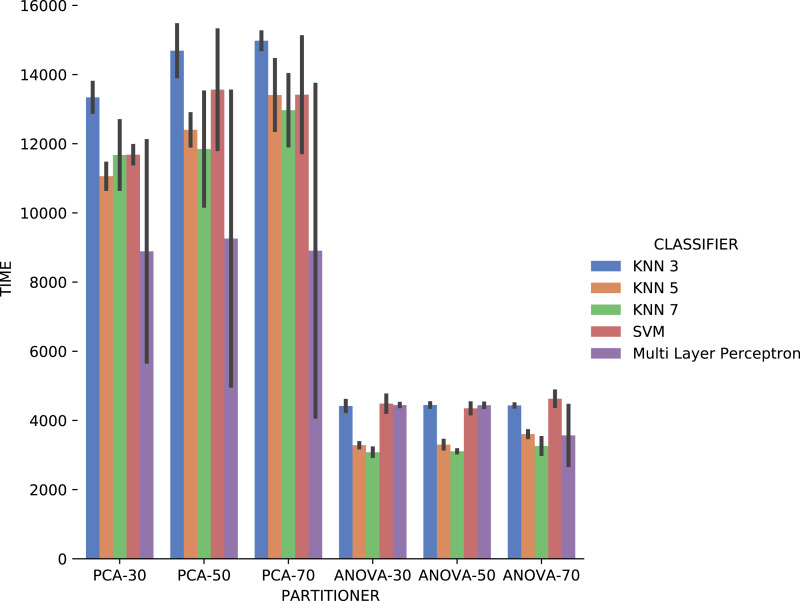
Experiment recommendation execution time with *Montage* data.

Concerning the results about the experiment time with categorical domain parameters from the *Montage* provenance database, presented in Fig. 23, one can see that the *KNN* classifier, *k* = 3, with *PCA* partitioning strategy was the most time-consuming. On the other hand, with the same *PCA* partitioning strategy, the *Multi Layer Perceptron* classifier used less time, but with a wide variation in recommendation times for different parameters. The *ANOVA* partitioning strategy continued to be a partitioning strategy that delivers the fattest recommendations. Still analyzing *ANOVA* partitioning strategy results, it is possible to see that the *KNN* classifier, with *k* ∈ {5,7}, was the fastest in recommending *Montage* workflow categorical domain parameters.

**Figure 24:**
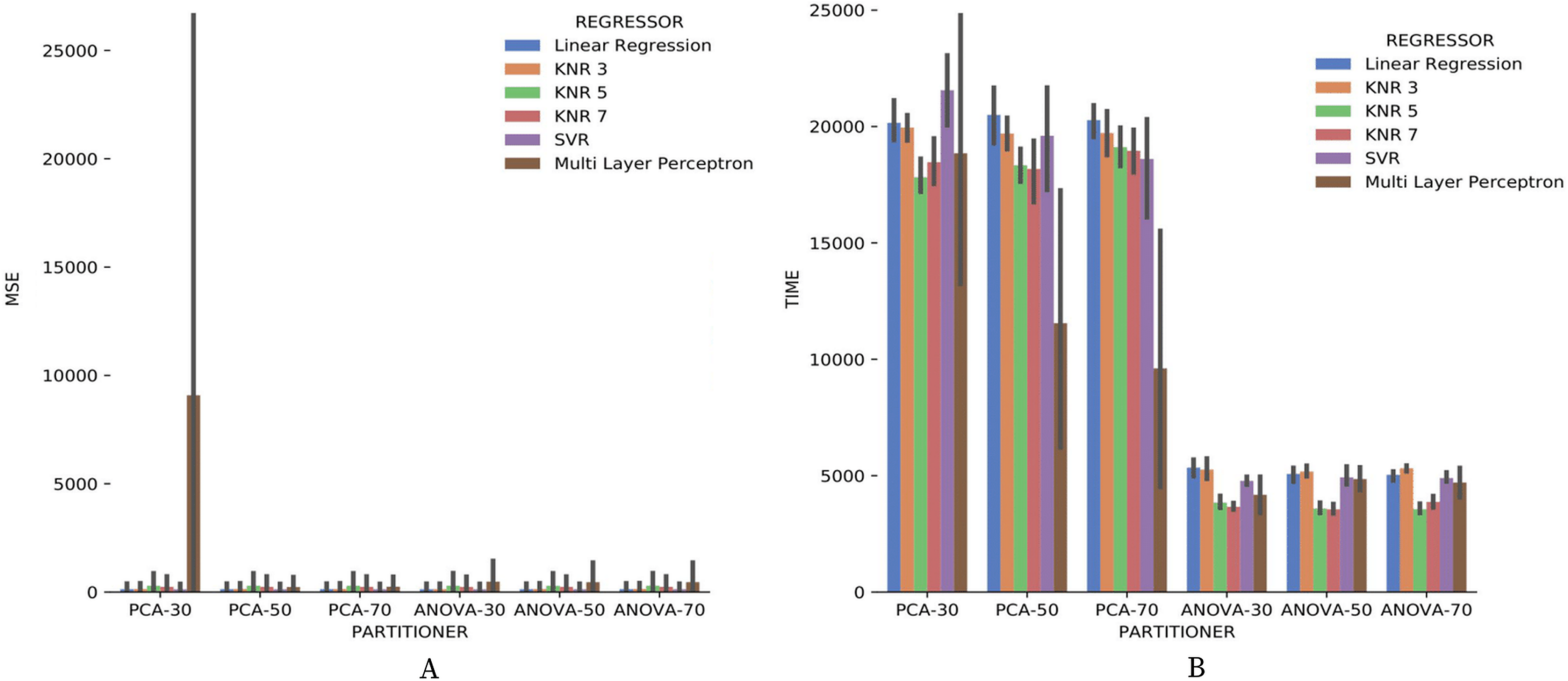
*MSE* results and recommendation execution time with *Montage* data.

Making a general analysis of results in Figs. 21, 22 and 23, the setup that uses *ANOVA* partitioning strategy with the *KNN* classifier, *k* = 7 it’s the best. This setup was the one that obtained the best results for *precision*, *recall*, and execution time spent simultaneously. *MSE* results for *Montage* numerical domain parameters presented in Fig. 24A show that, in general, the *MSE* was very close to zero for all cases, except in algorithm setup using *PCA* partitioning strategy with 30% elements in the generated partition and the *regressor* implemented by *Multi Layer Perceptron*. The *MSE* and its variation were very close to zero.

Regarding the execution time of Experiment 2 for numerical domain parameters recommendations for *Montage* data, Fig. 24B indicates the same behavior shown by results with *SciPhy* provenance database. Using *ANOVA* partitioning strategy and *KNR* regressors with *k* ∈ {5,7} as setup for Algorithm 2 produced the fastest recommendations.

The experiment execution time of *Montage* provenance database was much greater than the time used with the data from the workflow *Sciphy*. The explanation is the difference in the database size. Another observation is that the *ANOVA* partitioning strategy produces the fastest recommendations. Another point is that the percentage of the elements in partitioning generated by each partitioning strategy has no impact on the algorithm performance. Finally, it was possible to notice that the more robust *classifiers* and *regressors* had their performance exceeded by simpler models in some cases for the data used.

### Generic *FReeP* recommendation evaluation

A third experiment was modeled to evaluate Algorithm 3 performance. As in Experiment 2, different variations, following Table 4 values, were used in algorithm execution. *Precision*, *recall*, and *MSE* are also the metrics used to evaluate the recommendations made by each algorithm instance.

### Experiment 3. Algorithm 3 evaluation script

1. *n* Records from the provenance database were chosen as random examples.
2. *m* ≥ 2 random parameters were chosen for each example record as preferences, and their values are the same as those present in the example record.
3. Algorithm 3 was instantiated with a classifier or a regressor, a partitioning strategy, the partitions percentage to be returned by the partitioning strategy, and the selected *m* preferences.
4. Each returned recommendation is separated into numeric and categorical and is stored.
5. Precision and recall values were calculated for categorical recommendations and Mean Square Error (*MSE*) for numerical recommendations.

#### Results

Results showed here were obtained by fixing parameter *n* = 10 in Experiment 3, and using only *SciPhy* provenance database. Based on Experiment 2 results, it was decided to use the *ANOVA* partitioning strategy with 50% recovering elements from the provenance database. This choice is because the *ANOVA* partitioning strategy was the one that obtained the best results in previous experiment. As the percentage of data recovered by the strategy was not an impacting factor in the results, an intermediate percentage used in the previous experiment is selected. In addition, only *KNN*, with *k* ∈ {5,7}, and *SVM* were kept as classifiers, whereas only *KNR*, with *k* ∈ {5,7}, and *SVR* was chosen as regressors. These choices are supported by, in general, are the ones that present the best *precision*, *recall*, and *MSE* results in Experiment 2.

Table 5 presents the results obtained with the Algorithm 3 instance variations. Each row in the table represents an Algorithm 3 instance setup. The column that draws the most attention is the *Failures*. What happens is that, for some cases, the algorithm was not able to carry out the recommendation together and therefore did not return any recommendations. It is important to remember that each algorithm setup was tested on a set with 10 records extracted randomly from the database. The random record selection process can select records in which parameter values can be present only in the selected record. For this experiment, the selected examples are removed from the dataset, and therefore there is no other record that allows the correct execution of the algorithm.

**Table 5:** Experiment 3 results with Sciphy dataset.

Classifier	Regressor	Partitioning strategy	*MSE*	Precision	Recall	Failures
KNN 5	KNR 5	ANOVA 50	0.0	1.0	1.0	6
KNN 5	KNR 7	ANOVA 50	0.0	1.0	1.0	6
KNN 5	SVR	ANOVA 50	1.1075	1.0	1.0	6
KNN 7	KNR 5	ANOVA 50	4,279.2240	1.0	1.0	5
KNN 7	KNR 7	ANOVA 50	0.0	1.0	1.0	5
KNN 7	SVR	ANOVA 50	0.444	1.0	1.0	5
SVM	KNR 5	ANOVA 50	1,148.1876	0.75	0.75	6
SVM	KNR 7	ANOVA 50	0.0	1.0	1.0	7
SVM	SVR	ANOVA 50	0.0	1.0	1.0	7

Analyzing Table 5 results, focusing on the column *Failures* and taking into account that 10 records were chosen for each setup, it is possible to verify that in most cases, the algorithm was not able to make recommendations. However, considering only the recommendations made, it can be seen that the algorithm had satisfactory results for the precision and recall metrics. The values presented for the *MSE* metric were mostly satisfactory, differing only in the configurations of lines 4 and 7, both using the regressor *KNR* with *k* = 5. Another point to note is that the algorithm had more problems to make recommendations when the *SVM* classifier was used. Furthermore, it is possible to note that algorithm setups with more sophisticated Machine Learning models such as *SVM* and *SVR* do not add performance to the algorithm, specifically for *Sciphy* provenance dataset used.

## Related Work

Previous literature works had already relied on recommender systems to support scientific workflows. Moreover, hyperparameter tuning methods also have similar goals as paramater recommendation. Hyperparameters are variables that cannot be estimated directly from data, and, as a result, it is the user’s task to explore and define those values. *Hyperparameter Optimization (HPO)* is a research area that emerged to assist users in adjusting the hyperparameters of Machine Learning models in a non-ad-hoc manner (Yang & Shami, 2020). The well-defined processes resulting from research in the area may speed up the experimentation process and allow for reproducibility and fair comparison between models. Among the different methods of HPO, we can mention *Decision Theory*, *Bayesian Optimization*, *Multi-fidelity Optimization*, and *Metaheuristic Algorithms*.

Among the Decision Theory methods, the most used are *Grid Search* (Bergstra et al., 2011) and *Random Search* (Bergstra & Bengio, 2012). For both strategies, the user defines a list of values to be experimented for each hyperparameter. In Grid Search, the search for optimum values is given by experimenting the predefined values for the entire cartesian product. Random Search selects a sample for the hyperparameters to improve the execution time of the whole process. While the exponential search space of Grid Search may be impossible to complain, in Random Search there is the possibility that an optimal combination will not be explored. Also, the common problem between both approaches is that the dependencies between the hyperparameters are not taken into account. *FReeP* considers the possible dependencies between parameters by following the concept of classifier chains.

The Bayesian Optimization (Eggensperger et al., 2013) method optimizes the search space exploration using information from the previously tested hyperparameters to prune the non-promising combinations test. Despite using a surrogate model, the Bayesian optimization method still requires that the target model evaluation direct the search for the optimal hyperparameters. In a scenario of scientific workflows, it is very costly from the economical and runtime perspective to run an experiment, even more so to only evaluate a combination of parameter values. *FReeP* does not require any new workflow execution to recommend which values to use as it uses only data from past executions.

Multi-fidelity Algorithms (Zhang et al., 2016) also have the premise of balancing the time spent to search for hyperparameters. This kind of algorithm is based on successively evaluating hyperparameters in a subset search space. Those strategies follow similar motivations as the partitions generation of *FReeP*. However, in a scenario of scientific workflows, the Multi-fidelity algorithms still require workflow execution to evaluate combination quality.

The Metaheuristics Algorithms (Gogna & Tayal, 2013), based on the evolution of populations, use different forms of combinations of pre-existing populations in the hope of generating better populations at each generation. For hyperparameters tuning, hyperparameters with missing values are the population. Still, FReeP does not require any new execution of the workflow a priori to evaluate a recommendation given by the algorithm.

In general, the works that seek to assist scientists with some type of recommendation involving scientific workflow are focused on the composition phase. Zhou et al. (2018) uses a graph-based clustering technique to recommend workflows that can be reused in the composition of a developing workflow. De Oliveira et al. (2008) uses workflow provenance to extract connection patterns between components in order to make recommendations of new components for a workflow in composition. For each new component used in the composition of workflow, new components are recommended. Halioui, Valtchev & Diallo (2016), uses Natural Language Processing combined with specific ontologies in the field of Bioinformatics to extract concrete *workflows* from works in the literature. After the reconstruction of concrete *workflows*, tool combinations patterns, its parameters, and input data used in these *workflows* are extracted. All this data extracted can be used as assistance for composing new ones *workflows* that solve problems related to the mined *workflows*.

Yet concerned with assistance during the workflow composition phase, Mohan, Ebrahimi & Lu (2015) proposes the use of *Folksonomy* (Gruber, 2007) to enrich the data used for the recommendation of others *workflows* similar to a workflow under development. A design workflow tool was developed that allows free specification tags to be used in each component, making it possible to use not only the recommendation strategy through the workflow syntax, but also component semantics. Soomro, Munir & McClatchey (2015) uses domain ontologies as a knowledge base to incorporate semantics into the recommendation process. A hybrid recommender system was developed using ontologies to improve the already known recommendation strategy based on the extraction of standards from other *workflows*. Zeng, He & Van der Aalst (2011) uses data and control dependencies between activities, stored in the workflow provenance to build a causality table and another weights table. Subsequently, a *Petri network* (Zhou & Venkatesh, 1999) is used to recommend other components for the composition of workflow.

In the context of helping less experienced users in the use of scientific workflows, Wickramarachchi et al. (2018) and Mallawaarachchi et al. (2018) show experiments that prove that SWfMS *BioWorkflow* (Welivita et al., 2018) use is effective in increasing student engagement and learning in *Bioinformatics*.

Some works propose recommendation approaches that assist less experienced users in analysis of unknown domains, as is the case of Kanchana et al. (2016) and Kanchana et al. (2017), where a chart recommendation system was developed and evolved based on the use of metadata from any domain data. The system uses Machine Learning and Rule-based components that are refined with user feedback on the usefulness of the recommended charts.

Most of the approaches that uses recommender system methods to support the scientific process are closely linked to the experiment’s composition phase. The execution phase, where there is a need to adjust parameters, still lacks alternatives. Table 6 compares related work with *FReeP* approach. In Table 6 we show the name of the approach (column *Approach*), if it is focused on a specific domain or if its generic (column *Domain*), if it prunes the search space or considers the entire search space (column *Search Space*), if the approach considers dependencies among parameters (column *Considers Dependencies*), if it requires a new execution of the workflow or the application (column *Requires Execution*), and in which phase of the experiment life-cycle the approach is executed (column *Life-cycle Phase*). If there is no information about the analyzed characteristics in the paper we set as N/A (Not Available) in Table 6. This work proposes a hybrid recommendation algorithm capable of making value recommendations for one or multiple parameters of a *scientific workflow*, taking into account the user’s preferences.

**Table 6:** Comparison between FReeP and related work.

Approach	Domain	Search space	Considers dependencies	Requires execution	Life-cycle phase
Bergstra et al. (2011)	General	All	No	Yes	Execution
Bergstra & Bengio (2012)	General	Pruned	No	Yes	Execution
Eggensperger et al. (2013)	General	Pruned	No	Yes	Execution
Zhang et al. (2016)	General	Pruned	No	Yes	Execution
Gogna & Tayal (2013)	General	Pruned	No	Yes	Execution
Zhou et al. (2018)	Workflow	N/A	No	No	Composition
De Oliveira et al. (2008)	Workflow	N/A	No	No	Composition
Mohan, Ebrahimi & Lu (2015)	Workflow	N/A	No	No	Composition
Soomro, Munir & McClatchey (2015)	Workflow	N/A	No	No	Composition
Zeng, He & Van der Aalst (2011)	Workflow	N/A	Yes	No	Composition
Zhou & Venkatesh (1999)	Workflow	N/A	Yes	No	Composition
Wickramarachchi et al. (2018)	Workflow	N/A	N/A	N/A	Composition/Execution
Mallawaarachchi et al. (2018)	Workflow	N/A	N/A	N/A	Composition/Execution
Kanchana et al. (2016)	Workflow	N/A	N/A	No	Analysis
Kanchana et al. (2017)	Workflow	N/A	N/A	No	Analysis
**FReeP**	**Workflow**	**Pruned**	**Yes**	**No**	**Composition/Execution**

## Final Remarks

The *precision* and *recall* results obtained from the experiments suggest that *FReeP* is useful in recommending missing parameter values, decreasing the probability that failures will abort scientific experiments performed in High-Performance Computing environments. These results show a high-reliability degree, especially in the recommendation for one workflow parameter due to the number of experimental iterations performed to obtain the evaluations. The low availability of data for the experiments of the recommendation for *n* parameters impacts the reliability of the results obtained in this scenario. However, the results presented for the *n* parameters recommendation show that the approach is promising.

*FReeP* has a number of characteristics pointing out its contribution in saving runtime and financial resources when executing scientific experiments. First, *FReeP* can be executed on standard hardware, such as that used in the experiments presented in this article, without the need for an *HPC* environment. Besides, *FReeP* does not require any further execution of the scientific workflow to assess the recommendation’s quality as it uses provenance data. This characteristic of not requiring an instance of the scientific experiment to be performed is the huge difference and advantage compared with *Hyperparameter Optimization* strategies widely used in the Machine Learning models tuning.

In FReeP, all training data are collected and each tuple represents a different execution of the workflow. This data gathering process can nevertheless be time-consuming. However, one aspect that is expected is that the recommendation process will be performed once and a series of executions of the same workflow is repeated a significant number of (varying the known parameter). In addition, in many research groups there is already a database containing the provenance (Freire et al., 2008) that can be used to recommend parameter values for non-expert users, i.e., the scientists will not need to effectively execute the workflow to train the model since provenance data is already available. Public provenance repositories such as ProvStore (https://openprovenance.org/store/) (Huynh & Moreau, 2015) can be used as input for *FReeP*. For example, ProvStore contains 1,136 documents (each one associated with a workflow execution) of several different real workflows uploaded by research groups around the world.

From the perspective of runtime, when using the *ANOVA* partitioning strategy, in the experimental evaluation with the provenance data from the *Sciphy workflow*, the average time spent on the recommendations is about only *4 minutes*. In comparison, the average time of execution of the workflow *Sciphy* extracted from the provenance data used is about *17 h and 32 min*. Still taking into account the use of the *ANOVA* partitioning strategy, in the experimental evaluation with the *Montage* workflow provenance data, the average time spent on the recommendation is about *1 h and 30 min*. In contrast, the average execution time of a workflow experiment *Montage* extracted from the provenance data used was about *2 h and 3 min*.

Although it is more evident the lower relation between the experiment’s execution time and the recommendation time when analyzing the data from the *Sciphy* workflow, it is essential to emphasize that more robust hardware is not necessary to execute the recommendation process. Yet, future improvements in *FReeP* includes employing parallelism techniques to further decrease the recommendation time.

## Conclusion

The scientific process involves observing phenomena from different areas, formulating hypotheses, testing, and refining them. Arguably, this is an arduous job for the scientist in charge of the process. With the advances in computational resources, there is a growing concern about helping scientists in scientific experimentation. A significant step towards a more robust aid was the adoption of *scientific workflows* as a model for representing scientific experiments and *Scientific Workflow Management Systems* to support the management of experiment executions.

Computational execution of the experiments represented as *scientific workflows* relies on the use of computer programs that play the role of each stage of the experiment. In addition to input data, these programs often need additional configuration parameters to be adjusted to simulate the experiment’s conditions. The scientist responsible for the experiment ends up developing an intuition about the sets of parameters that lead to satisfactory results. However, another scientist who runs the same experiment will not have the same experience, which may lead him/her to define a set of parameters that will not result in a successful experiment.

Several proposals in the literature have aimed at supporting the composition phase of the experiments, but recommending parameter values for the experiment execution phase is still an open field. This article presented *FReeP: Feature Recommender From Preferences*, an algorithm for recommending values for parameters in *scientific workflows* considering the user’s preferences. The goal was to allow a new user to express their preferences of values for a subset of workflow parameters and recommend values for the parameters that had no preference defined. FReeP has three versions, all of them relying on Machine Learning techniques. Two approaches focused on the value recommendation for one parameter at a time. The third instance addresses recommending values for all the other parameters of a workflow for which a user preference was not defined.

The proposed algorithm proved to be useful for recommending one parameter, indicating a path for the recommendation of *n* parameters. Nevertheless, there are some limitations. *FReeP*, as a memory-based algorithm, faces scalability issues as its implementation can consume a lot of computational resources. Yet, the recommendations of *FReeP* are limited to the existence of examples on the provenance dataset. This means that the algorithm cannot make any “default” recommendations if there are no examples for the algorithm’s execution or recommend values that are not present in the provenance dataset. Also, the recommendation algorithm may have a longer processing time than the experiment itself. Another point is that all the instances have the same weight during the recommendation process. The algorithm does not consider the user’s expertise that performed the previous execution to adjust an example’s weight. Still, the algorithm considers only the set of parameters of the workflow; however, a set of parameters may be more or less relevant according to the input data. Additionally, the recommendation algorithm may end up recommending a set of values present in the provenance base that causes a workflow execution failure.

Based on those limitations, there are some proposals for future work. First proposal is parallelizing the processing of the generated partitions, which should decrease the time spent on the recommendation. In addition, evaluating *FReeP* on data from other domains and evaluating the tradeoff between the recommendation time and the algorithm execution time. Also, associating weights with examples from the provenance dataset according to the user’s profile. Lastly, using instances from the provenance dataset that failed to execute the workflow as a constraint to improve the recommendations’ results.

## Supplemental Information

10.7717/peerj-cs.606/supp-1 Supplemental Information 1 Provenance data extracted from actual executions of the Montage workflow. One part was run using SciCumulus Workflow Management System, the other part retrieved from the Workflow Generator site (https://confluence.pegasus.isi.edu/display/pegasus/WorkflowGenerator).
The crota2 attribute is a float value that represents an image rotation on the sky. The dec attribute is an optional float representing Dec for region statistics, and crval2 is a float value representing Axis 2 sky reference value in Montage workflow.

10.7717/peerj-cs.606/supp-2 Supplemental Information 2 Provenance data extracted from actual executions of the workflow SciPhy performed using SciCumulus Workflow Management System. The attribute prob1 is the probability that a given evolutive relationship is valid. Prob2 attribute is the probability that a given evolutive relationship
is valid. The num aligns attribute is the total number of alignments in a given data file. Finally, the length attribute is the maximum sequence length in a specific data file.

## Additional Information and Declarations

### Data Availability

The following information was supplied regarding data availability: All the code and data are available at https://github.com/MeLL-UFF/FReeP. The CSV files are available in the Supplemental Files.

## References

[ref-1] Adomavicius G, Tuzhilin A (2005). Toward the next generation of recommender systems: a survey of the state-of-the-art and possible extensions. IEEE Transactions on Knowledge and Data Engineering.

[ref-2] Al-Sharrah G (2010). Ranking using the copeland score: a comparison with the hasse diagram. Journal of Chemical Information and Modeling.

[ref-3] Altintas I, Ludaescher B, Klasky S, Vouk MA (2006). Introduction to scientific workflow management and the kepler system.

[ref-4] Bergeron C, Zaretzki J, Breneman C, Bennett KP (2008). Multiple instance ranking.

[ref-5] Bergstra JS, Bardenet R, Bengio Y, Kégl B (2011). Algorithms for hyper-parameter optimization.

[ref-6] Bergstra J, Bengio Y (2012). Random search for hyper-parameter optimization. Journal of Machine Learning Research.

[ref-7] Bose R, Foster I, Moreau L (2006). Report on the international provenance and annotation workshop: (ipaw’06) 3–5 May 2006, Chicago. ACM SIGMOD Record.

[ref-8] Bottou L (2010). Large-scale machine learning with stochastic gradient descent.

[ref-9] Burke R (2002). Hybrid recommender systems: survey and experiments. User Modeling and User-Adapted Interaction.

[ref-10] Coates A, Ng AY (2011). The importance of encoding versus training with sparse coding and vector quantization.

[ref-11] De Oliveira D, Ogasawara E, Baião F, Mattoso M (2010a). Scicumulus: a lightweight cloud middleware to explore many task computing paradigm in scientific workflows.

[ref-12] De Oliveira DCM, Liu J, Pacitti E (2019). Data-intensive workflow management: for clouds and data-intensive and scalable computing environments. Synthesis Lectures on Data Management.

[ref-13] De Oliveira FT, Murta L, Werner C, Mattoso M (2008). Using provenance to improve workflow design. International Provenance and Annotation Workshop.

[ref-14] De Oliveira D, Ocaña KACS, Baião FA, Mattoso M (2012). A provenance-based adaptive scheduling heuristic for parallel scientific workflows in clouds. Journal of Grid Computing.

[ref-15] De Oliveira D, Ocaña KACS, Ogasawara E, Dias J, Gonçalves J, Baião F, Mattoso M (2013). Performance evaluation of parallel strategies in public clouds: a study with phylogenomic workflows. Future Generation Computer Systems.

[ref-16] De Oliveira D, Ogasawara E, Baião F, Mattoso M (2010b). Scicumulus: a lightweight cloud middleware to explore many task computing paradigm in scientific workflows.

[ref-17] Deelman E, Singh G, Su M-H, Blythe J, Gil Y, Kesselman C, Mehta G, Vahi K, Berriman G, Good J, Laity A, Katz DS (2005). Pegasus: a framework for mapping complex scientific workflows onto distributed systems. Scientific Programming.

[ref-18] Eggensperger K, Feurer M, Hutter F, Bergstra J, Snoek J, Hoos H, Leyton-Brown K (2013). Towards an empirical foundation for assessing bayesian optimization of hyperparameters.

[ref-19] Emerson P (2013). The original Borda count and partial voting. Social Choice and Welfare.

[ref-20] Freire J, Koop D, Santos E, Silva CT (2008). Provenance for computational tasks: a survey. Computing in Science & Engineering.

[ref-21] Fürnkranz J, Hüllermeier E (2003). Pairwise preference learning and ranking.

[ref-22] Fürnkranz J, Hüllermeier E, Sammut C, Webb GI (2011). Preference learning. Encyclopedia of Machine Learning.

[ref-23] Garthwaite PH, Jolliffe IT, Jolliffe I, Jones B (2002). Statistical inference.

[ref-24] Gil Y, Miles S, Belhajjame K, Deus H, Garijo D, Klyne G, Missier P, Soiland-Reyes S, Zednik S (2013). Prov model primer: W3C working group note. https://global.oup.com/academic/product/statistical-inference-9780198572268?cc=br&lang=en&.

[ref-25] Girden ER (1992). ANOVA: repeated measures—number 84.

[ref-26] Goble C (2002). Position statement: musings on provenance, workflow and (semantic web) annotations for bioinformatics.

[ref-27] Gogna A, Tayal A (2013). Metaheuristics: review and application. Journal of Experimental & Theoretical Artificial Intelligence.

[ref-28] Gonçalves B, Porto F (2015). Managing scientific hypotheses as data with support for predictive analytics. Computing in Science & Engineering.

[ref-29] Gruber T (2007). Ontology of folksonomy: a mash-up of apples and oranges. International Journal on Semantic Web and Information Systems.

[ref-30] Guedes T, Jesus LA, Ocaña KACS, Drummond LMA, De Oliveira D (2020a). Provenance-based fault tolerance technique recommendation for cloud-based scientific workflows: a practical approach. Cluster Computing.

[ref-31] Guedes T, Martins LB, Falci MLF, Silva V, Ocaña KACS, Mattoso M, Bedo M, De Oliveira D (2020b). Capturing and analyzing provenance from spark-based scientific workflows with SAMbA-RaP. Future Generation Computer Systems.

[ref-32] Guerine M, Stockinger MB, Rosseti I, Simonetti LG, Ocaña KACS, Plastino A, De Oliveira D (2019). A provenance-based heuristic for preserving results confidentiality in cloud-based scientific workflows. Future Generation Computer Systems.

[ref-33] Halioui A, Valtchev P, Diallo AB (2016). Towards an ontology-based recommender system for relevant bioinformatics workflows. bioRxiv.

[ref-34] Herlocker JL, Konstan JA, Terveen LG, Riedl JT (2004). Evaluating collaborative filtering recommender systems. ACM Transactions on Information Systems.

[ref-35] Hey T, Gannon D, Pinkelman J (2012). The future of data-intensive science. Computer.

[ref-36] Hey T, Trefethen AE (2020). The fourth paradigm 10 years on. Informatik Spektrum.

[ref-37] Hoffa C, Mehta G, Freeman T, Deelman E, Keahey K, Berriman B, Good J (2008). On the use of cloud computing for scientific workflows.

[ref-38] Huynh TD, Moreau L, Ludäscher B, Plale B (2015). Provstore: a public provenance repository. Provenance and Annotation of Data and Processes.

[ref-39] Hüllermeier E, Fürnkranz J, Cheng W, Brinker K (2008). Label ranking by learning pairwise preferences. Artificial Intelligence.

[ref-40] Jacob JC, Katz DS, Berriman GB, Good JC, Laity A, Deelman E, Kesselman C, Singh G, Su M-H, Prince T, Williams R (2009). Montage: a grid portal and software toolkit for science-grade astronomical image mosaicking. International Journal of Computational Science and Engineering.

[ref-41] Kanchana W, Madushanka G, Maduranga H, Udayanga M, Meedeniya D, Perera G (2016). Context aware recommendation for data visualization.

[ref-42] Kanchana W, Madushanka G, Maduranga H, Udayanga M, Meedeniya D, Perera I (2017). Semi-automated recommendation platform for data visualization: Roopana.

[ref-43] Karvonen L (2004). Preferential voting: incidence and effects. International Political Science Review.

[ref-44] Keller JM, Gray MR, Givens JA (1985). A fuzzy k-nearest neighbor algorithm. IEEE Transactions on Systems, Man, and Cybernetics.

[ref-45] Kohavi R (1995). A study of cross-validation and bootstrap for accuracy estimation and model selection. Proceedings of the 14th international joint conference on Artificial intelligence - Volume 2 (IJCAI’95).

[ref-46] Lestari S, Adji TB, Permanasari AE (2018). Performance comparison of rank aggregation using borda and copeland in recommender system.

[ref-47] Lika B, Kolomvatsos K, Hadjiefthymiades S (2014). Facing the cold start problem in recommender systems. Expert Systems with Applications.

[ref-48] Mallawaarachchi V, Wickaramarachchi A, Weliwita A, Perera I, Meedeniya D (2018). Experiential learning in bioinformatics—learner support for complex workflow modelling and analysis. International Journal of Emerging Technologies in Learning.

[ref-49] Marozzo F, Talia D, Trunfio P (2013). Scalable script-based data analysis workflows on clouds.

[ref-50] Mattoso M, Werner C, Travassos GH, Braganholo V, Murta L, Ogasawara E, De Oliveira D, Da Cruz SMS, Martinho W (2010). Towards supporting the life cycle of large-scale scientific experiments. International Journal of Business Process Integration and Management.

[ref-51] McKinney W (2011). Pandas: a foundational python library for data analysis and statistics.

[ref-52] Mitchell TM (2015). Machine learning.

[ref-53] Mohan A, Ebrahimi M, Lu S (2015). A folksonomy-based social recommendation system for scientific workflow reuse.

[ref-54] Myers RH, Myers RH (1990). Classical and modern regression with applications.

[ref-55] Nie Z, Zhang Y, Wen J-R, Ma W-Y (2005). Object-level ranking: bringing order to web objects.

[ref-56] Ocaña KA, De Oliveira D, Ogasawara E, Dávila AM, Lima AA, Mattoso M (2011). Sciphy: a cloud-based workflow for phylogenetic analysis of drug targets in protozoan genomes.

[ref-57] Ocaña KACS, Galheigo M, Osthoff C, Gadelha LMR, Porto F, Gomes ATA, De Oliveira D, Vasconcelos AT (2020). Bioinfoportal: a scientific gateway for integrating bioinformatics applications on the brazilian national high-performance computing network. Future Generation Computer Systems.

[ref-58] Ogasawara E, De Oliveira D, Valduriez P, Dias J, Porto F, Mattoso M (2011). An algebraic approach for data-centric scientific workflows. Proceedings of the VLDB Endowment.

[ref-59] Pedregosa F, Varoquaux G, Gramfort A, Michel V, Thirion B, Grisel O, Blondel M, Prettenhofer P, Weiss R, Dubourg V (2011). Scikit-learn: machine learning in python. Journal of Machine Learning Research.

[ref-60] Rani P, Shokeen J, Mullick D (2017). Recommendations using modified k-means clustering and voting theory. International Journal of Computer Science and Mobile Computing.

[ref-61] Read J, Pfahringer B, Holmes G, Frank E (2011). Classifier chains for multi-label classification. Machine Learning.

[ref-62] Resnick P, Varian HR (1997). Recommender systems. Communications of the ACM.

[ref-63] Ricci F, Rokach L, Shapira B, Ricci F, Rokach L, Shapira B, Kantor P (2011). Introduction to recommender systems handbook. Recommender Systems Handbook.

[ref-64] Schein AI, Popescul A, Ungar LH, Pennock DM (2002). Methods and metrics for cold-start recommendations.

[ref-65] Silva Junior D, Paes A, Pacitti E, De Oliveira D (2018). Freep: towards parameter recommendation in scientific workflows using preference learning.

[ref-66] Silva V, Neves L, Souza R, Coutinho ALGA, De Oliveira D, Mattoso M (2020). Adding domain data to code profiling tools to debug workflow parallel execution. Future Generation Computer Systems.

[ref-67] Soomro K, Munir K, McClatchey R (2015). Incorporating semantics in pattern-based scientific workflow recommender systems: improving the accuracy of recommendations.

[ref-68] Tang Y, Tong Q (2016). Bordarank: a ranking aggregation based approach to collaborative filtering.

[ref-69] Taylor AD, Pacelli AM (2008). Mathematics and politics: strategy, voting, power, and proof.

[ref-70] Tsoumakas G, Katakis I (2007). Multi-label classification: an overview. International Journal of Data Warehousing and Mining.

[ref-71] Van der Walt S, Colbert SC, Varoquaux G (2011). The numpy array: a structure for efficient numerical computation. Computing in Science & Engineering.

[ref-72] Vembu S, Gärtner T (2011). Label ranking algorithms: a survey.

[ref-73] Viappiani P, Boutilier C (2009). Regret-based optimal recommendation sets in conversational recommender systems.

[ref-74] Wang L (2005). Support vector machines: theory and applications.

[ref-75] Welivita A, Perera I, Meedeniya D, Wickramarachchi A, Mallawaarachchi V (2018). Managing complex workflows in bioinformatics: an interactive toolkit with gpu acceleration. IEEE Transactions on Nanobioscience.

[ref-76] Wickramarachchi A, Mallawaarachchi V, Meedeniya D, Perera I, Welivita A (2018). Enhanced student learning in proteomics-an interactive tool support for teaching workflows.

[ref-77] Yang L, Shami A (2020). On hyperparameter optimization of machine learning algorithms: theory and practice. Neurocomputing.

[ref-78] Zeng R, He X, Van der Aalst WM (2011). A method to mine workflows from provenance for assisting scientific workflow composition.

[ref-79] Zhang S, Xu J, Huang E, Chen C-H (2016). A new optimal sampling rule for multi-fidelity optimization via ordinal transformation.

[ref-80] Zhao Y, Ioan Raicu IF (2008). Scientific workflow systems for 21st century, new bottle or new wine?.

[ref-81] Zhou Z, Cheng Z, Zhang L-J, Gaaloul W, Ning K (2018). Scientific workflow clustering and recommendation leveraging layer hierarchical analysis. IEEE Transactions on Services Computing.

[ref-82] Zhou M, Venkatesh K (1999). Modeling, simulation, and control of flexible manufacturing systems: a Petri net approach.

